# Obesity Is Associated with an Impaired Baseline Repertoire of Anti-Influenza Virus Antibodies

**DOI:** 10.1128/spectrum.00010-23

**Published:** 2023-04-26

**Authors:** Marwa Abd Alhadi, Lilach M. Friedman, Erik A. Karlsson, Liel Cohen-Lavi, Anat Burkovitz, Stacey Schultz-Cherry, Terry L. Noah, Samuel S. Weir, Lester M. Shulman, Melinda A. Beck, Tomer Hertz

**Affiliations:** a Department of Microbiology, Immunology and Genetics, Faculty of Health Sciences, Ben-Gurion University of the Negev, Beer-Sheva, Israel; b National Institute for Biotechnology in the Negev, Ben-Gurion University of the Negev, Beer-Sheva, Israel; c Virology Unit, Institute Pasteur du Cambodge, Phnom Penh, Cambodia; d Department of Industrial Engineering and Management, Ben-Gurion University of the Negev, Beer-Sheva, Israel; e Department of Infectious Diseases, St. Jude Children’s Research Hospital, Memphis, Tennessee, USA; f Department of Pediatrics, University of North Carolina at Chapel Hill, Chapel Hill, North Carolina, USA; g Department of Family Medicine, University of North Carolina at Chapel Hill, Chapel Hill, North Carolina, USA; h Department of Epidemiology and Preventive Medicine, School of Public Health, Sackler Faculty of Medicine, Tel Aviv University, Tel Aviv, Israel; i Department of Nutrition, Gillings School of Global Public Health, University of North Carolina, Chapel Hill, North Carolina, USA; j Vaccine and Infectious Disease Division, Fred Hutchinson Cancer Research Center, Seattle, Washington, USA; Utrecht Institute for Pharmaceutical Sciences, Utrecht University

**Keywords:** antibody repertoire, influenza vaccines, obesity

## Abstract

Obesity is a risk factor for severe disease and mortality for both influenza and severe acute respiratory syndrome coronavirus 2 (SARS-CoV-2) infection. While previous studies show that individuals with obesity generate antibody responses following influenza vaccination, infection rates within the obese group were twice as high as those in the healthy-weight group. The repertoire of antibodies raised against influenza viruses following previous vaccinations and/or natural exposures is referred to here as baseline immune history (BIH). To investigate the hypothesis that obesity impacts immune memory to infections and vaccines, we profiled the BIH of obese and healthy-weight adults vaccinated with the 2010-2011 seasonal influenza vaccine in response to conformational and linear antigens. Despite the extensive heterogeneity of the BIH profiles in both groups, there were striking differences between obese and healthy subjects, especially with regard to A/H1N1 strains and the 2009 pandemic virus (Cal09). Individuals with obesity had lower IgG and IgA magnitude and breadth for a panel of A/H1N1 whole viruses and hemagglutinin proteins from 1933 to 2009 but increased IgG magnitude and breadth for linear peptides from the Cal09 H1 and N1 proteins. Age was also associated with A/H1N1 BIH, with young individuals with obesity being more likely to have reduced A/H1N1 BIH. We found that individuals with low IgG BIH had significantly lower neutralizing antibody titers than individuals with high IgG BIH. Taken together, our findings suggest that increased susceptibility of obese participants to influenza infection may be mediated in part by obesity-associated differences in the memory B-cell repertoire, which cannot be ameliorated by current seasonal vaccination regimens. Overall, these data have vital implications for the next generation of influenza virus and SARS-CoV-2 vaccines.

**IMPORTANCE** Obesity is associated with increased morbidity and mortality from influenza and SARS-CoV-2 infection. While vaccination is the most effective strategy for preventing influenza virus infection, our previous studies showed that influenza vaccines fail to provide optimal protection in obese individuals despite reaching canonical correlates of protection. Here, we show that obesity may impair immune history in humans and cannot be overcome by seasonal vaccination, especially in younger individuals with decreased lifetime exposure to infections and seasonal vaccines. Low baseline immune history is associated with decreased protective antibody responses. Obesity potentially handicaps overall responses to vaccination, biasing it toward responses to linear epitopes, which may reduce protective capacity. Taken together, our data suggest that young obese individuals are at an increased risk of reduced protection by vaccination, likely due to altered immune history biased toward nonprotective antibody responses. Given the worldwide obesity epidemic coupled with seasonal respiratory virus infections and the inevitable next pandemic, it is imperative that we understand and improve vaccine efficacy in this high-risk population. The design, development, and usage of vaccines for and in obese individuals may need critical evaluation, and immune history should be considered an alternate correlate of protection in future vaccine clinical trials.

## INTRODUCTION

Obesity is widely spread globally, with over 1.9 billion adults (39%) that are overweight and more than 650 million (14%) obese adults. It is estimated that over 2.8 million people die due to complications related to overweight and obesity annually. While obesity is associated with higher risks of chronic diseases, recent studies have reported associations between obesity and an increased risk of infectious diseases (1, 2). During the 2009 A/H1N1 pandemic, obesity was recognized as an independent risk factor for complications from influenza, including disease caused by seasonal and emerging influenza virus strains (3). The ongoing severe acute respiratory coronavirus 2 (SARS-CoV-2) pandemic further highlights the role of obesity as an independent risk factor for severe outcomes from respiratory virus infections (4–6). The expansive prevalence of obesity worldwide, coupled with significant influenza mortality, even in nonpandemic years (7–9), makes identifying factors that affect influenza vaccination outcomes in the obese population a critical need. Although vaccination is the primary method of influenza prevention, adults with obesity vaccinated against influenza have an increased risk of influenza infection or influenza-like illness compared to vaccinated nonobese adults, despite generating what is generally considered a seroprotective response (10). To further understand how obesity may impact antibody responses to influenza vaccination, we conducted an in-depth analysis of influenza-specific IgG and IgA antibody repertoires of 205 participants, 100 with healthy weights (HW group; body mass index [BMI] = 18.5 to 24.9 kg/m^2^) and 105 with obesity (obese group; BMI ≥ 30 kg/m^2^) prior to and 30 days after vaccination with a split-virus trivalent influenza vaccine, which was the first to include the A/H1N1 2009 (Cal09) pandemic strain (2010-2011). Using a variety of different influenza virus antigens, including whole viruses, recombinant hemagglutinin (HA) proteins, and peptides that span the HA and neuraminidase (NA) proteins of the Cal09 strain, we found that the repertoire of antibodies raised against influenza viruses following previous vaccinations and/or natural exposures, referred to here as baseline immune history (BIH), and postvaccination responses were significantly different in individuals with obesity and healthy-weight controls, especially with regard to A/H1N1 influenza virus strains. We further showed that while both obese and healthy-weight participants respond to influenza vaccination, vaccination does not compensate for the differences in baseline immune history of the two groups.

## RESULTS

### BIH to the A/H1N1 influenza subtype is significantly lower in individuals with obesity.

**FIG 1:**
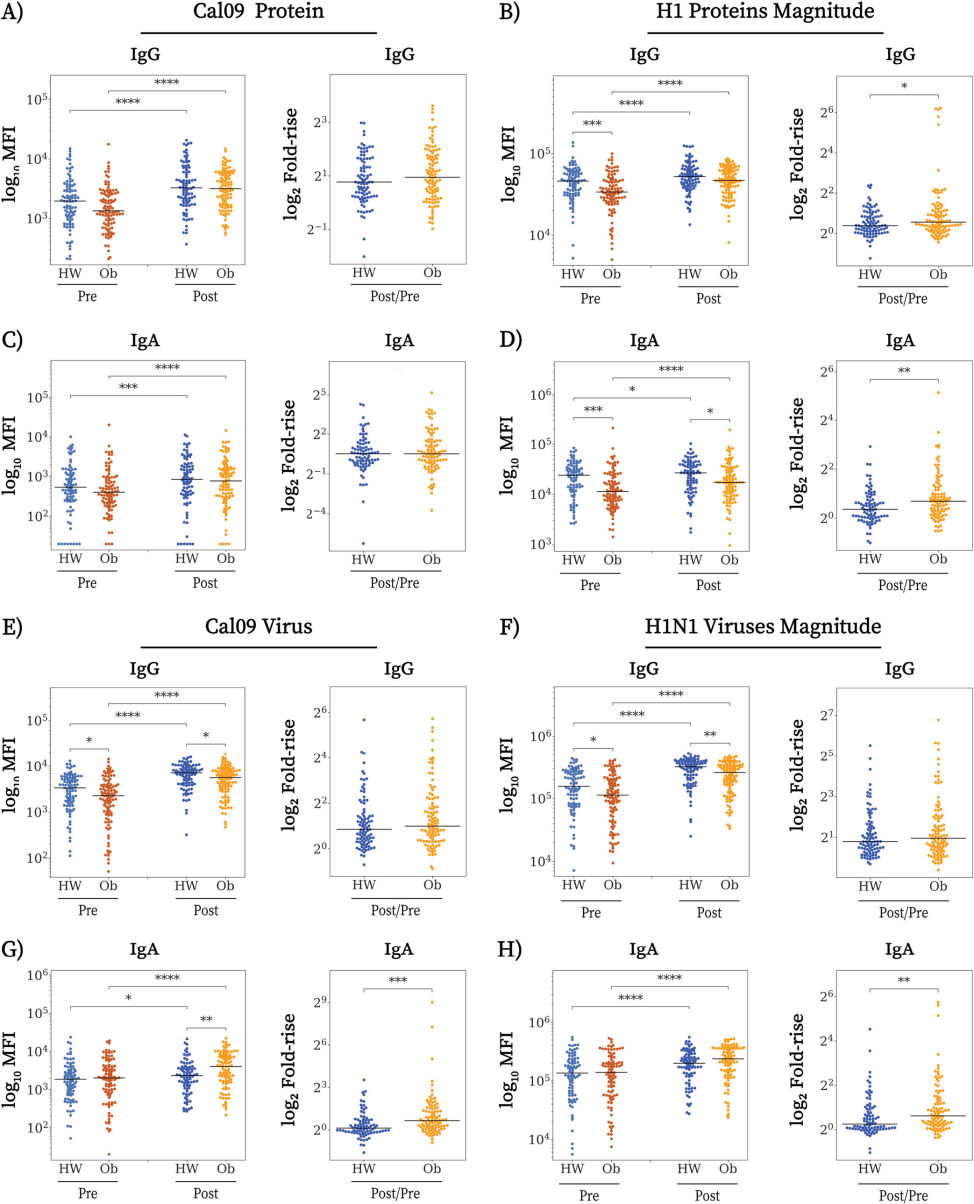
Obese individuals have altered baseline immune history to influenza H1N1. Baseline and postvaccination antibody levels and fold rise in 89 healthy-weight (HW) and 100 obese (Ob) individuals to a panel of 8 H1N1 HA (H1) proteins and 11 H1N1 BPL-inactivated viruses spotted on antigen microarrays (Table S1). (A) IgG binding to the HA of H1N1 Cal09. (B) IgG magnitude to a panel of 8 H1 proteins. (C) IgA binding to the HA of Cal09. (D) IgA magnitude to a panel of 8 H1 proteins. (E) IgG binding Cal09 BPL-inactivated virus. (F) IgG magnitude to a panel of 11 H1N1 viruses. (G) IgA binding to Cal09 BPL-inactivated virus. (H) IgA magnitude to a panel of 11 H1N1 viruses. Lines represent the MFI, the boxes denote the 25th and 75th percentiles, and the error bars represent 1.5 times the interquartile range. Statistical significance was assessed using the Wilcoxon signed-rank test (baseline versus postvaccination) and the Wilcoxon rank-sum test (HW versus obese). *, *P* < 0.05; **, *P* < 0.005; ***, *P* < 0.0005; ****, *P* < 0.00005.

Serum samples were obtained from 100 obese and 105 HW human participants prevaccination (baseline) and 30 days after vaccination with the 2010-2011 trivalent inactivated influenza vaccine (TIV) that included the A/H1N1/California/7/2009 (Cal09), A/H3N2/Perth/16/2009 (Perth09), and B/Brisbane/60/2008 (Brisbane08) strains (see Materials and Methods). All samples were normalized to a total protein concentration of 10 mg/mL as measured by Nanodrop. BIH varies between individuals (11–13). Therefore, we first measured the prevaccination BIH for IgG and IgA responses to HA proteins from the Cal09, Perth09, and Brisbane08 vaccine strains, and 20 additional seasonal vaccine and historical influenza strains from 1933 to 2016 (listed in Table S1 in the supplemental material), which were spotted on microarrays. While baseline IgG levels against Cal09 HA were marginally lower in the obese group (*P* = 0.050) (Fig. 1A), baseline IgG levels against HA antigens of Perth09 and Brisbane08 were similar in obese and HW individuals (Fig. S1A and S2A). At baseline, individuals with obesity had a significantly lower magnitude (sum of levels of antibody to all strains from a given subtype) and breadth (number of strains to which a participant has antibodies) for IgG against HA proteins of A/H1N1 (Fig. 1B; Fig. S3A) and lower magnitude of IgG to B HA (Fig. S2B) but not to A/H3N2 strains (Fig. S1B). There were no differences in BIH IgA responses between HW and obese adults to HAs of the 3 TIV strains (Fig. 1C; Fig. S1C and S2C) or in the BIH IgA magnitude and breadth for A/H3N2 or B proteins in the panel (Fig. S1D and S2D [magnitude] and Fig. S3B [breadth]). However, the magnitude and breadth of the baseline IgA repertoire against HA proteins of A/H1N1 strains were significantly lower in individuals with obesity, similarly to baseline IgG levels (Fig. 1D; Fig. S3B, vaccination values).

We also measured BIH antibody responses against 34 whole inactivated viruses from 1933 to 2017 (Table S1). Interestingly, individuals with obesity had significantly lower baseline levels of IgG to all 3 TIV strains (Fig. 1E; Fig. S1E and S2E) and had significantly decreased IgG magnitude (Fig. 1F; Fig. S1F and S2F) and IgG breadth (Fig. S3C) to all 3 subtypes in the panel prevaccination. In contrast, there were no significant differences in baseline IgA levels between obese and HW individuals for the current strains (Fig. 1G; Fig. S1G and S2G) and no differences in magnitude (Fig. 1H; Fig. S1H and S2H) and breadth (Fig. S3D) of IgA responses to whole viruses.

Taken together, these observations show that clear differences in baseline responses exist between obese and HW individuals: the obese group had lower prevaccination levels of IgG against the vaccine strains, decreased breadth and magnitude of IgG to 34 strains of whole influenza viruses, and decreased magnitude and breadth of IgA against HA proteins from 8 historical A/H1N1 strains of influenza virus. Thus, prior to vaccination, obese participants displayed a diminished antibody response against influenza virus, despite multiple exposures and vaccinations, in particular for subtype A/H1N1.

### Vaccination elicits immune responses in both obese and healthy-weight individuals.

While individuals with obesity displayed decreased BIH, previous studies showed that they do respond to influenza vaccination (seroconversion and seroprotection) at rates similar to those in healthy-weight individuals (10, 14). Therefore, to determine whether seasonal vaccination might overcome the obesity-associated BIH deficit in the short term, we measured serum antibody responses at 28 to 35 days postvaccination. Postvaccination IgG and IgA levels significantly increased in both HW and individuals with obesity against the HA of Cal09 (Fig. 1A and C) and Perth09 (Fig. S1A and C) vaccine strains but not to Brisbane08 (Fig. S2A and C). Both groups also exhibited significantly increased magnitude and breadth of both IgG and IgA to HAs from all three subtypes (Fig. 1B and D; Fig. S1B and D, S2B and D, and S3A and B). However, vaccine-induced increases in magnitude of both IgG and IgA against A/H1N1 HA proteins and IgA against B type HA proteins were significantly higher in the individuals with obesity (Fig. 1B and D; Fig. S2D). Nevertheless, postvaccination magnitudes of IgG to A/H1N1 HAs were similar in both groups (Fig. 1B), and IgA magnitudes were lower in the obese group due to the lower IgA magnitude at baseline (Fig. 1D). Postvaccination increases in magnitude of IgA or IgG against A/H3N2 strains or IgG against B strains were not significantly different between HW and individuals with obesity (Fig. S1B and D and S2B).

In terms of whole virus antigens, a significant rise in IgG and IgA levels against all isolates postvaccination was observed in both obese and HW individuals. However, comparing between groups, postvaccination IgG levels to the three vaccine strains and magnitude of responses to the three subtypes were significantly lower in individuals with obesity (Fig. 1E and F; Fig. S1E and F and S2E and F). Differences in postvaccination IgG breadth were noted only for A/H3N2 strains (Fig. S3C). In individuals with obesity, the lower anti-influenza virus IgG levels postvaccination were possibly driven by the lower BIH IgG levels, as the vaccine-induced fold change in IgG levels, breadth, and magnitude were similar between obese and HW individuals (Fig. 1F to H; Fig. S1F to H and S2F to H). In contrast, levels of IgA to vaccine strains, as well as magnitude and breadth, were significantly higher in the individuals with obesity postvaccination, due to stronger vaccine-induced IgA responses in individuals with obesity, as reflected by the significantly higher fold change in IgA to whole influenza viruses (Fig. 1G and H; Fig. S1G and H and S2G and H).

### IgG baseline immune history is associated with neutralizing antibody responses.

**FIG 2:**
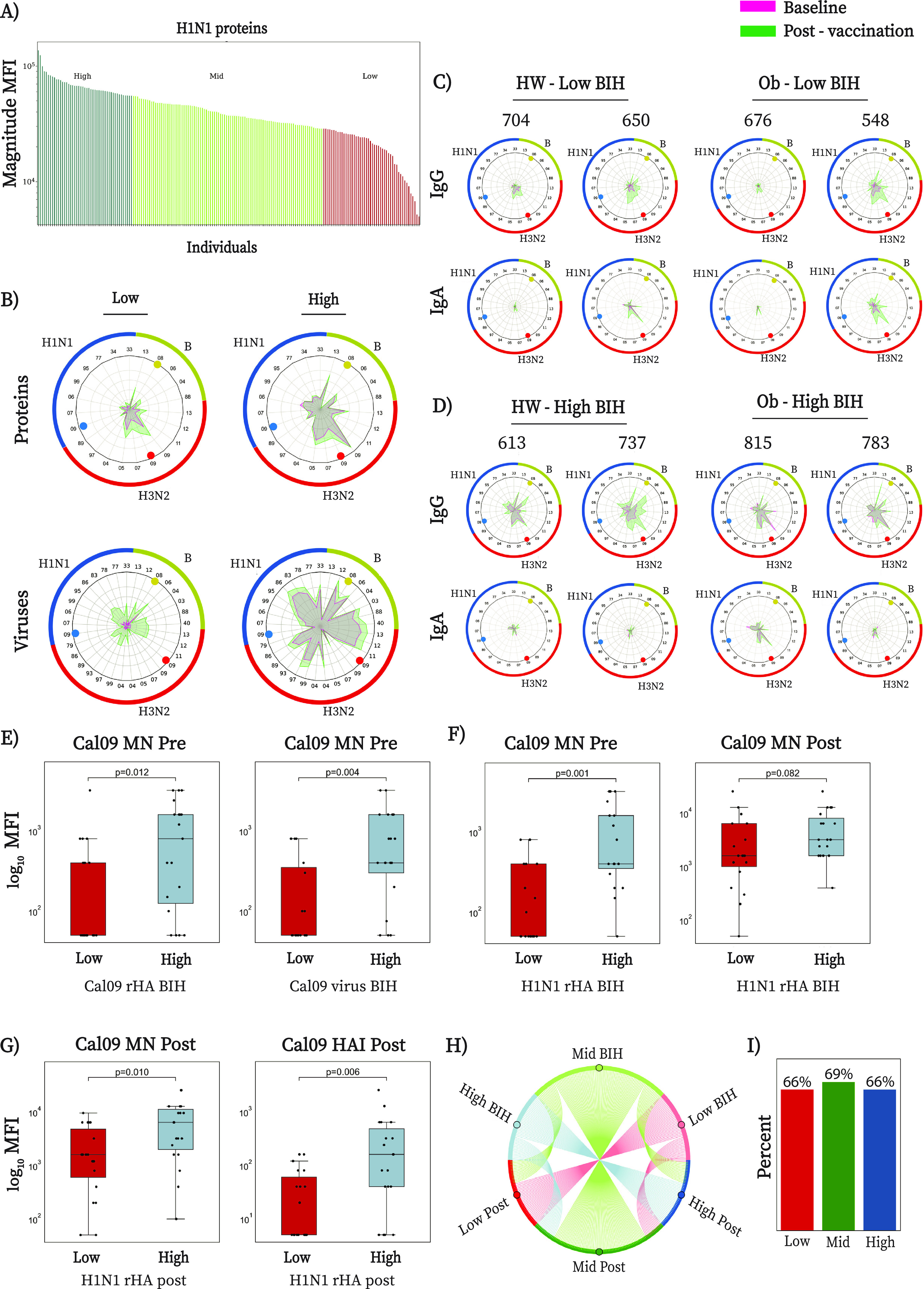
Baseline immune history IgG profiles are heterogeneous and are associated with neutralizing antibody responses. (A) Ranking a cohort of 189 individuals (89 HW and 100 obese) by IgG BIH to HA magnitude against a panel of 8 H1N1 strains (H1 proteins). Quartiles are used to define the low-BIH (green), mid-BIH (yellow), and high-BIH (red) groups. (B) Spider plots of the average normalized IgG response in the low- and high-BIH groups, sorted by magnitude to H1 proteins (top) and H1N1 viruses (bottom). Each vertex represents the normalized binding of IgG to a single influenza antigen. Baseline and postvaccination antibody repertoires are denoted by magenta and green, respectively. The numbers on the outer circles denote the year each strain was isolated, and the three vaccine strains are denoted by the colored dots. The upper spider plots present average antibody repertoires to HA proteins of (counterclockwise) A/H1N1 strains from 1933 to 2009 (blue segment of the circle), A/H3N2 strains from 1989 to 2013 (red segment of the circle), and B strains from 1988 to 2013 (green segment of the circle). The lower spider plots present average antibody repertoires to virus isolates that appear in the order listed in Table S1. (C and D) IgG and IgA spider plots for 4 representative low-BIH (C) and 4 representative high-BIH (D) individuals, as sorted by the baseline IgG magnitudes to H1 proteins. From each quartile, data for two HW and two obese individuals are presented. The binding results for each antigen were normalized to the maximal binding result of this antigen across all the samples. (E) Box plots comparing baseline Cal09 MN titers of the low-BIH and high-BIH groups ranked by IgG to Cal09 H1 protein (left) or Cal09 whole-virus antigen (right). (F) Box plots comparing baseline (left) and postvaccination (right) Cal09 MN titers of the low- BIH and high-BIH groups ranked by IgG magnitude to H1 proteins. (G) Box plots comparing postvaccination MN titers (left) and HAI titers (right) in the low-response versus high-response groups ranked by postvaccination IgG magnitude to H1 proteins. (H) Transition matrix from BIH groups to postvaccination response groups using chord plots for 76 individuals. Each line represents a single individual and connects that individual’s BIH group and postvaccination response (Post) group. Lines are colored according to the BIH group: pink, low BIH; green, mid-BIH; blue, high BIH. (I) Percentage of individuals that remain in their BIH group postvaccination. Panels E to I present results from a subgroup of 76 individuals recruited in the first study year (2010-2011) for which MN and HAI titers were measured. Statistical significance was assessed using the Wilcoxon rank sum test. *, *P* < 0.05; **, *P* < 0.005; ***, *P* < 0.0005.

BIH differs for each individual depending on previous exposure and vaccine history. Indeed, extensive BIH heterogeneity was observed in both IgG and IgA baseline magnitude and breadth against influenza virus antigens, including HA proteins, whole viruses, and peptides (for examples, see Fig. 1). To further study the heterogeneity of immune history and its effect on vaccine-induced responses, all baseline samples were blindly ranked by decreasing BIH IgG or IgA magnitudes of response to whole virus or HA A/H1N1 antigens. For each baseline marker, the cohort was divided into quartiles: individuals in the lowest quartile were designated the low-BIH group, individuals in the highest quartile were designated the high-BIH group, and the remaining two middle quartiles were designated the mid-BIH group (for example, see Fig. 2A for IgG H1 ranking). Each quartile included both obese and HW individuals. To visualize the BIH of each participant, we generated spider plots for the mean BIH profile of each group. For example, spider plots of the mean IgG responses were plotted for the low and high quartiles based on IgG BIH to H1 proteins (Fig. 2B, top), and spider plots of the mean IgG repertoire were plotted for the low and high quartiles based on IgG BIH to A/H1N1 viruses (Fig. 2B, bottom). Spider plots were also generated for a representative subset of obese and HW low-BIH and high-BIH individuals based on IgG BIH to H1 proteins (Fig. 2C and D), further highlighting that each individual has a unique BIH profile that varies in both overall magnitude and breadth but also in the specificity to each subtype and to individual strains within the panel. Interestingly, some participants with low IgG BIH to A/H1N1 HA proteins also had low IgG BIH to A/H3N2 and B HA proteins (e.g., participants 704 and 676) (Fig. 2C), while others had higher levels of IgG to other subtypes (e.g., participant 548). We found that the antibody response to the vaccine was heavily biased by the individual BIH. While some low-BIH participants failed to respond to the vaccine (e.g., participant 676), others generated robust vaccine-induced immune responses (e.g., participants 650 and 548). A comparison between the IgG and IgA spider plots for each participant demonstrated that most participants with low IgG BIH to H1 also had low IgA BIH to H1. However, in some cases, there were striking differences between IgG and IgA profiles (e.g., participants 737 and 783) (Fig. 2D).

To study whether ranking individuals by BIH was associated with microneutralization (MN) and hemagglutination inhibition (HAI) titers, which are canonical correlates of protection for influenza (15–18), we ranked a subset of 76 individuals recruited during year 1 of the study (2010) for which Cal09 MN titers were measured at baseline and postvaccination and HAI titers were measured postvaccination. To assess the functional relevance of IgG BIH ranking, we compared the Cal09 neutralization titer of individuals in the low-BIH versus high-BIH groups. We found that individuals with low IgG BIH to the Cal09 HA protein or virus had significantly lower baseline Cal09 MN titers than individuals with high IgG BIH (Fig. 2E). Similarly, individuals with low IgG BIH to A/H1N1 HA proteins or viruses also had significantly lower baseline MN titers (Fig. 2F, left). Furthermore, individuals with lower IgG BIH to A/H1N1 HA proteins tended to also have lower neutralizing titers postvaccination, but these associations were not statistically significant (*P* = 0.082) (Fig. 2F, right). Ranking individuals by postvaccination magnitudes of responses to A/H1N1 HA proteins was significantly associated with postvaccination MN titers, as well as with postvaccination HAI titers (Fig. 2G). IgA BIH and postvaccination rankings were not associated with MN or HAI titers.

### Baseline immune history biases postvaccination immune responses.

Given that IgG BIH to H1 proteins is associated with MN antibody titers at baseline and that postvaccination IgG responses to H1 proteins are also associated with postvaccination MN and HAI responses, we next asked to what extent individuals transition from one response group to another. We found that 31/47 (66%) of individuals within the A/H1N1 HA IgG low-BIH group remained within the low-response group postvaccination (Fig. 2H, Low Post, and Fig. 2I), with similar rates for obese and HW individuals. Similar findings were also observed for the mid-BIH and high-BIH groups (Fig. 2H and I). This was further supported by the significant correlation between the baseline and postvaccination IgG and IgA antibody titers to H1 proteins (IgG *r* = 0.76; IgA *r* = 0.86) (Fig. S4A and C). Therefore, while vaccination significantly increased IgG and IgA antibody responses, in both obese and HW participants, the overall ranking of individuals based on antibody responses to A/H1N1 HA antigens was not significantly shifted postvaccination. Similar trends were also observed for the A/H1N1 viruses IgG BIH rankings, but the correlation between BIH and postvaccination rankings were weaker overall (IgG *r* = 0.60; IgA *r* = 0.78) (Fig. S4B and D).

### Individuals with obesity have increased IgG antibody responses to linear influenza virus epitopes.

**FIG 3:**
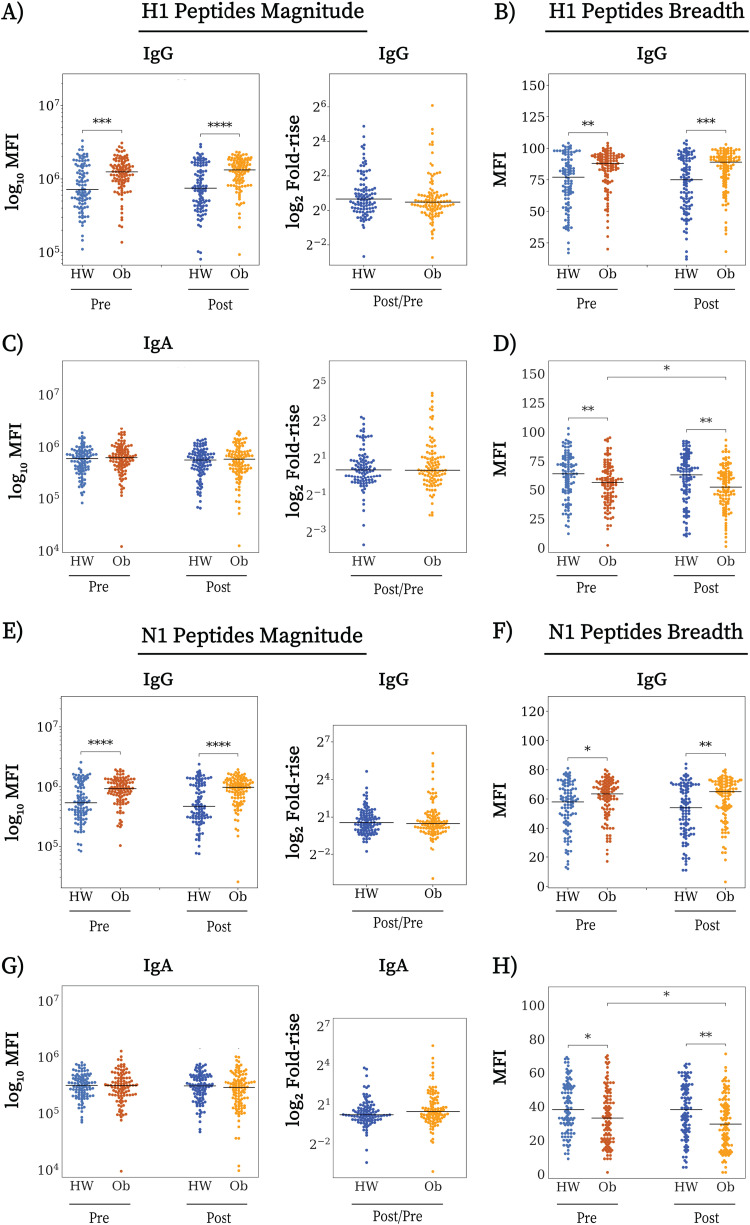
Obese individuals have increased IgG BIH to peptides from H1 and N1 of the Cal09 vaccine strain. Arrays spotted with 20-mer amino acid peptides spanning the HA and NA proteins of the H1N1 vaccine strain (15-aa overlap) were used to profile the repertoires of IgG and IgA responses to linear epitopes of Cal09. Responses were summarized using magnitude (the sum of responses to all peptides of the same protein) and breadth (the number of peptides from each protein to which the participant had antibodies). (A) IgG H1 magnitude and fold rise; (B) IgG H1 breadth; (C) IgA H1 magnitude and fold rise; (D) IgA H1 breadth; (E) IgG N1 magnitude and fold rise; (F) IgG N1 breadth; (G) IgA N1 magnitude and fold rise; (H) IgA N1 breadth. Lines represent the MFI, the boxes denote the 25th and 75th percentiles, and the error bars represent 1.5 times the interquartile range. Statistical significance was assessed using the Wilcoxon signed-rank test (prevaccination versus postvaccination) and the Wilcoxon rank-sum test (HW versus Ob). *, *P* < 0.05; **, *P* < 0.005; ***, *P* < 0.0005.

Since antibodies can target linear epitopes in addition to conformational epitopes, we next characterized the BIH and postvaccination IgG and IgA repertoire using peptide microarrays spotted with a succession of 20-mer peptides with partial overlap of 15 amino acids (aa) spanning the HA and NA protein sequences (H1 and N1) of the A/H1N1 Cal09 vaccine strain. We computed the magnitude and breadth of responses to peptides from the H1 and N1 proteins (see Materials and Methods). There was an inverse correlation between recognition of linear epitopes of peptides and recognition of conformational epitopes on whole viruses and HAs for IgG. Specifically, pre- and postvaccination sera from individuals with obesity had a higher magnitude and broader repertoire of IgG antibodies to Cal09 H1 and N1 peptides (Fig. 3A, B, E, and F), in contrast to their lower recognition of Cal09 whole virus (Fig. 1E) and H1 proteins (Fig. 1B). In contrast, prevaccination IgA breadth, but not magnitude, to Cal09 H1 and N1 peptides was significantly lower in individuals with obesity (Fig. 3C, D, G, and H), as well as prevaccination IgA breadth to H1 proteins (Fig. S3B) and postvaccination IgA breadth to A/H1N1 viruses (Fig. S3D). While postvaccination IgA response to whole A/H1N1 viruses and HA proteins was stronger in the obese group, leading to a higher postvaccination IgA level to whole Cal09 virus (Fig. 1D and G), this phenomenon was not observed in the IgA repertoire to Cal09 peptides, and postvaccination IgA breadth to Cal09 H1 and N1 peptides remained lower in the obese group (Fig. 3D and H). These findings suggest that an increase in the level of IgG antibodies to conformational antigens is associated with a decrease in the diversity and quantity of IgG antibodies to linear peptides. This association was not observed for IgA. The differences between the obese and HW antibody response described here were specific to influenza virus antigens, since the total IgG and IgA levels in the sera of the two groups were similar, as detected by enzyme-linked immunosorbent assay (ELISA) (Fig. S5).

### Baseline immune history to influenza virus is associated with BMI and age.

**FIG 4:**
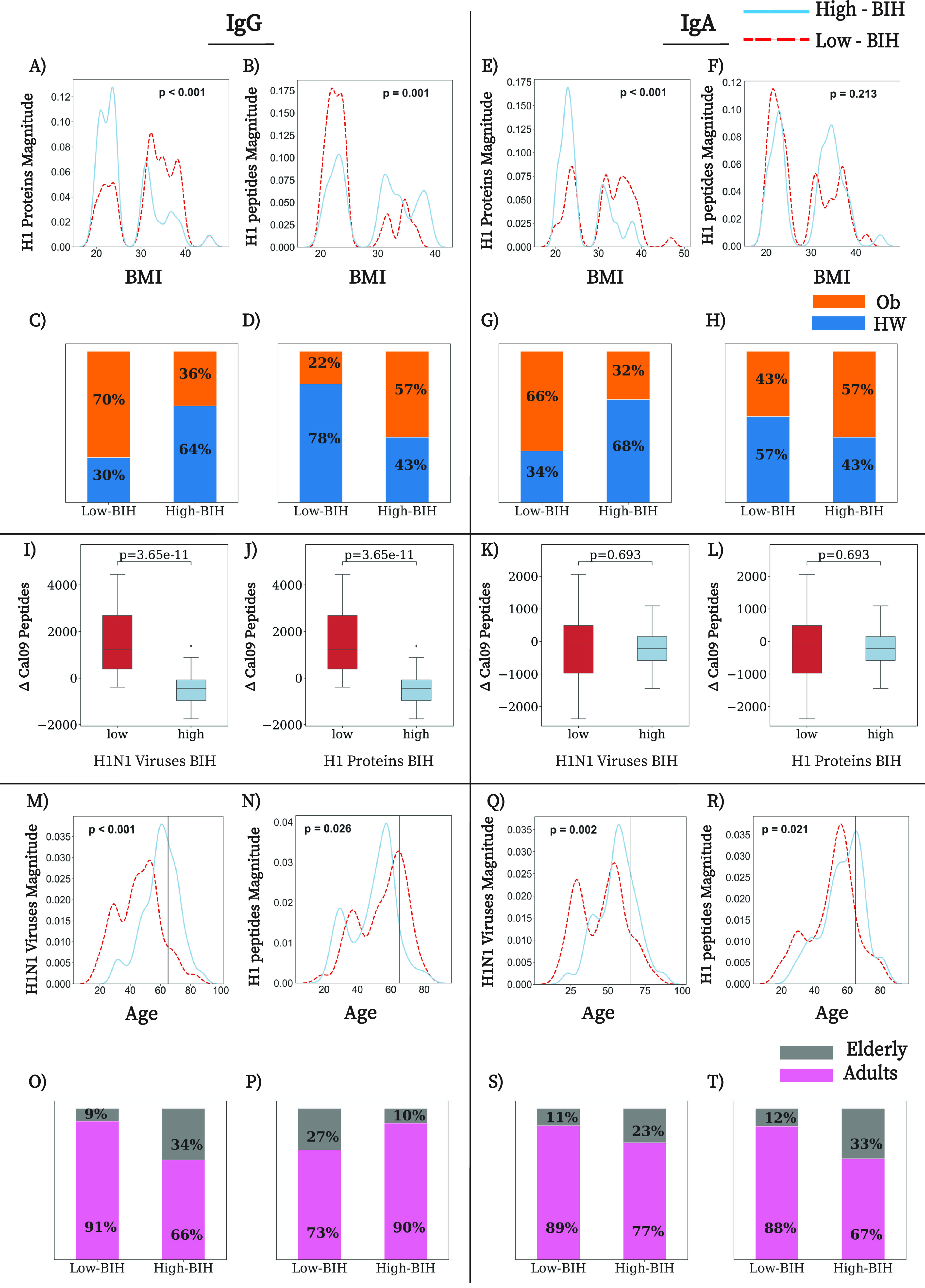
BMI and age are associated with BIH. (A to H) Obesity was associated with low IgG BIH to H1N1 viruses and H1 proteins but high IgG BIH to peptides of the Cal09 H1 and N1 proteins. (A and B) Distributions of BMI in the low-BIH groups (dashed red line) and the high-BIH groups (solid blue line) ranked by (A) IgG magnitude against H1N1 proteins and (B) IgG magnitude to H1 peptides. (C and D) Percentages of obese (orange) and HW (blue) participants in the low- and high-BIH groups for (C) IgG magnitude to H1N1 proteins and (D) IgG magnitude to H1 peptides. (E) IgA magnitude to H1N1 proteins. (F) IgA magnitude to H1 peptides. (G and H) Percentages of obese participants in the low- and high-BIH groups for (G) IgA magnitude against H1N1 proteins and (H) IgA magnitude to H1 peptides. Overweight participants (25 < BMI < 30) were excluded from our analysis. (I to L) Box plots of the average IgG (I and J) and IgA (K and L) responses to Cal09 H1 and N1 peptides in low- and high-BIH groups sorted by H1 proteins and H1N1 viruses. In each plot, we compare the distribution of the baseline-subtracted antibody responses to Cal09 H1 and N1 peptides following vaccination in the low-BIH (red) and high-BIH (blue) groups ranked by a specific marker. Only peptides in which there was a >4-fold change (rise or decline) in antibody titer postvaccination were selected. (I) Postvaccination IgG responses to Cal09 peptides ranked by IgG H1N1 virus magnitude; (J) postvaccination IgG responses to Cal09 peptides ranked by IgG H1 magnitude; (K) postvaccination IgA responses to Cal09 peptides ranked by IgA H1N1 viruses magnitude; (L) postvaccination IgA responses to Cal09 peptides ranked by IgA H1 proteins magnitude. (M to T) Old age (>65 years) was associated with increased IgG and IgA BIH to H1N1 viruses and H1 proteins but reduced IgG and IgA BIH to H1 and N1 peptides. (M and N) Distributions by age group comparing adult (<65) and elderly individuals within the low-BIH group and the high-BIH groups ranked by (M) IgG magnitude against H1N1 viruses and (N) IgG magnitude to H1 peptides. (O and P) Percentages of elderly (>65 years old, gray) individuals in the low- and high-BIH groups sorted by (O) IgG magnitude against H1N1 viruses and (P) IgG magnitude to H1 peptides. (Q) IgA magnitude to H1N1 viruses. (R) IgA magnitude to H1 peptides. (S and T) Percentages of elderly (>65 years old) individuals in the low- and high-BIH groups for (S) IgA magnitude to H1N1 viruses and (T) IgA magnitude to H1 peptides. *P* values comparing the differences between the BMI (A to H) or age (M to T) distributions of the low-BIH and high-BIH groups were determined using the Wilcoxon rank sum test. The numbers of participants in each low- and high-BIH group are listed in Table 3.

To further study the association between obesity and baseline immune history to A/H1N1, we compared the BMI distributions of the low-BIH and high-BIH groups ranked by IgG and IgA responses to viruses, proteins and peptides (Fig. 4A to H; Fig. S6A to H). A significantly higher frequency of individuals with obesity was present in the low-BIH group for magnitude of IgG antibodies to both A/H1N1 viruses (relative risk [RR], 1.86; 95% confidence interval [CI], 1.19 to 2.91) and H1 proteins (RR, 2.07; 95% CI, 1.29 to 3.34) (Fig. 4A and C; Fig. S6A and C and S7A). A significantly higher frequency of individuals with obesity was also present in the low-BIH group for magnitude of IgA antibodies to H1 proteins (RR, 2.02; 95% CI, 1.27 to 3.21) but not to A/H1N1 viruses (RR, 1.10; 95% CI, 0.72 to 1.69) (Fig. 4E and G; Fig. S6E and G and S7A). Similar findings were also observed for IgG BIH groups to influenza A/H3N2 and influenza B whole-virus antigens, as well as B HA proteins, but not for IgA BIH groups of A/H3N2 and B antigens (Fig. S8A to H, S9A to H, and S7A). In contrast, when the individuals were ranked according to IgG binding to H1 and N1 peptides, individuals with obesity were significantly less frequent in the low-BIH groups for H1 magnitude (RR, 0.43; 95% CI, 0.25 to 0.73) and N1 magnitude (RR, 0.43; 95% CI, 0.25 to 0.73) (Fig. 4B and D; Fig. S6B and D and S7A). Similar findings were also observed for rankings based on breadth of responses to H1 and N1 peptides (Fig. S7 and S10A to D). No significant associations were found for IgA-based peptide rankings (Fig. 4E to H; Fig. S7A, S6E to H, and S10E to H). The inverse relationship between IgG responses to conformational and linear epitopes can also be observed when comparing the postvaccination response to H1 and N1 peptide antigens in the low- and high-IgG-BIH groups ranked by H1 proteins or A/H1N1 viruses (Fig. 4I to L). Specifically, we found that individuals with low IgG BIH to H1 proteins or A/H1N1 viruses had increased antibody responses to Cal09 peptides postvaccination and individuals in the high-IgG-BIH groups had decreased antibody responses to these peptides (Fig. 4I and J). No significant differences were found when comparing IgA responses to peptides in the low- and high-IgA-BIH groups for A/H1 proteins and A/H1N1 viruses (Fig. 4K and L).

We used the same approach to compare the age distribution of individuals in the low- and high-BIH groups. We found a significantly lower frequency of elderly individuals (>65) in the low-BIH group for magnitude of IgG antibodies to A/H1N1 viruses (RR, 2.91; 95% CI, 1.18 to 7.13) but not to H1 proteins (Fig. 4M and O; Fig. S7B and S6I and K). Similar trends were also observed for A/H3N2 and B viruses (Fig. S8I and K and S9I and K). This contrasted with higher frequencies of elderly persons in the low IgG-BIH magnitude groups for H1 peptides (RR, 0.56; 95% CI, 0.39 to 0.79) and N1 peptides (RR, 0.55; 95% CI, 0.39 to 0.78) (Fig. 4N and P; Fig. S7B and S6J and L), as well as for both breadth groups (Fig. S10I to L). IgA-based rankings of both A/H1N1 viruses and A/H1 proteins were also associated with age (Fig. 4Q and S; Fig. S6M and O and S7B), with a lower frequency of elderly persons in the low-BIH groups. However, in contrast to IgG, there was also a significantly lower number of elderly persons in the lower-IgA-BIH quartiles for IgA magnitudes to H1 and N1 peptides (RR, 2.18; 95% CI, 1.07 to 4.46 for both) (Fig. 4R and T; Fig. S6N and P and S7B). Thus, unlike IgG responses, there was no significant inverse correlation between magnitude of IgA antibodies to conformational (viruses and proteins) and linear (peptides) antigens when weight or age was taken into account.

We then checked whether younger and older individuals with obesity differ by their BIH to A/H1N1 antigens, since both obese individuals and adults were enriched in the low-IgG-BIH groups for whole A/H1N1 viruses and in the high-IgG-BIH groups for H1 and N1 peptides, as well as in the low-IgA-BIH groups for H1 proteins (Fig. 4). While no correlation was found between age and BMI (*P* = 0.85), the age distributions of individuals with obesity in the low-BIH and high-BIH groups were compared. Individuals with obesity in the groups with low IgG and low IgA BIH to whole viruses were significantly younger than individuals with obesity in the groups with high BIH to viruses (IgG *P* = 0.000001; IgA *P* = 0.002). Furthermore, individuals with obesity in the group with low IgA BIH to H1 proteins were younger than the individuals with obesity in the group with high IgA BIH to proteins (*P* = 0.02), while the age of individuals with obesity in groups with IgG BIH to H1 proteins was similar (*P* = 0.9). No similar age differences were found when HW individuals from the low-BIH and high-BIH groups were compared.

### IgG and IgA antibodies of obese and HW individuals target different functional sites in the Cal09 HA protein at baseline.

**FIG 5:**
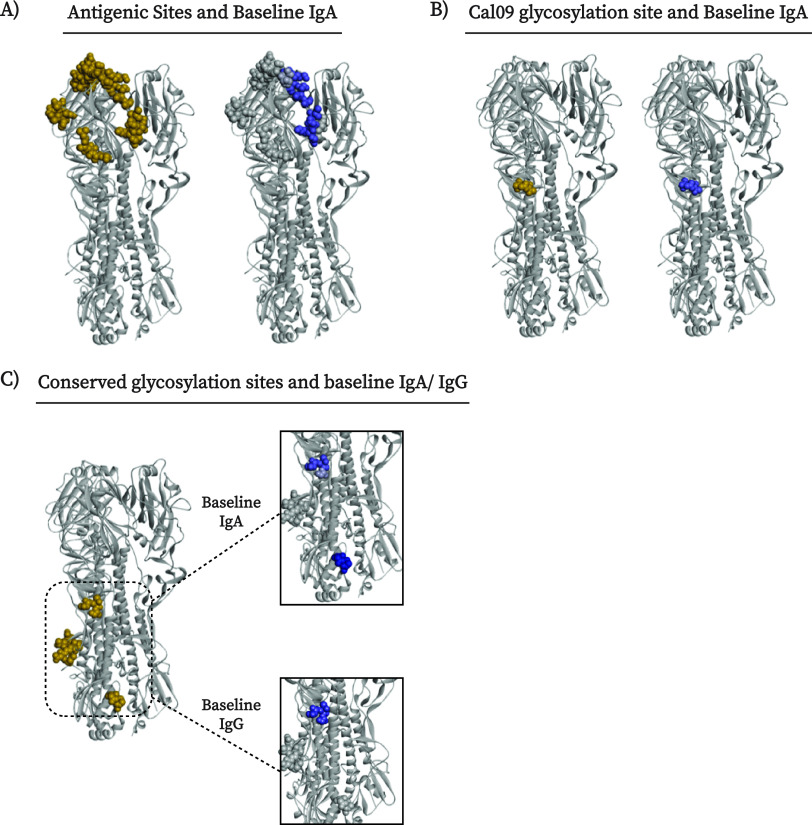
Cal09 HA protein domains are differentially targeted by BIH antibodies of obese and healthy-weight individuals. A logistic regression model was trained to discriminate between HW and obese individuals using the IgG and IgA antibody profiles to the HA peptides of the Cal09 vaccine strain. The weights assigned by the model were used to score individual amino acids on the HA protein based on the maximal weight of a given position across all of the peptides in which it was included (see Materials and Methods for details). Figures were created using Discovery Studio Visualizer software and the crystal structure of the Cal09 HA trimeric protein (PDB ID 3LZG) (46). Gold spheres represent amino acids differentially targeted by obese individuals, and blue spheres those preferentially targeted by healthy-weight individuals within (A) antigenic sites, (B) the Cal09 glycosylation site, and (C) conserved glycosylation sites. Panels A and B present positions differentially targeted by BIH IgA, and panel C presents both IgG and IgA responses.

The significant differences between obese and HW that were found in the magnitude and breadth of both IgG and IgA antibodies to Cal09 H1 peptides prompted us to ask whether different peptides were targeted in the obese and HW individuals. We mapped the H1 peptides targeted by IgG and IgA antibodies of these BIH groups to the structure of the Cal09 HA protein, and epitopes targeted preferentially by obese and HW individuals were compared using a logistic regression model. Relative scaled values were assigned to responses to individual peptides, and the score for an individual position was based on the maximal absolute value assigned to the peptides that overlapped that position. We found that HW IgG antibodies preferentially targeted positions in the HA1 subunit and a conserved glycosylation site, while only IgG from obese individuals targeted the more conserved HA2 subunit (Table 1; Fig. 5C; Fig. S11A and B). Of the eight functional domains analyzed, five (receptor binding site [RBS], esterase domain, conserved glycosylation sites, Cal09 glycosylation site, and the antigenic sites) were preferentially targeted by IgA of HW individuals, while only the fusion domain was preferentially targeted by IgA antibodies from individuals with obesity (Table 1; Fig. 5; Fig. S11C to E). These findings further demonstrate that the differences between the HW and obese individuals can be observed even at the resolution of specific functional domains, which may have functional implications for providing protection against infection and development of disease.

**TABLE 1:** Targeting of HA functional sites by obese and HW individuals^*a*^

Site	IgA				IgG			
	Baseline level in group		No. of residues	*P*	Baseline level in group		No. of residues	*P*
	Obese	HW			Obese	HW		
HA protein	85	114	498		59	40	498	
HA1	50	65	322	0.7438	34	40	322	1 × 10e−4**
HA2	35	48	174	0.3591	23	0	174	0.0003**
Fusion	76	53	261	<1 × 10e−4**	33	20	261	0.3008
RBS	0	20	153	0.0509	20	20	153	0.9176
Esterase	7	26	64	0.0006**	0	0	64	0.8066
Conserved glycosylation sites	0	6	13	0.0152**	0	3	13	0.0255**
Cal09 glycosylation sites	0	3	3	0.0131**	0	0	3	0.8293
Antigenic sites	0	11	50	0.0371**	5	0	50	0.1896

a **, *P* < 0.005.

## DISCUSSION

The SARS-CoV-2 pandemic has echoed the influenza virus A/H1N1 2009 pandemic in identifying obesity as a risk factor for severe disease and mortality (5, 6, 9, 19–22). Several recent studies reported diminished antibody responses in individuals with obesity following coronavirus disease 2019 (COVID-19) vaccination (23, 24). The current study investigated the hypothesis that the differences in vaccine-induced protection between obese and HW individuals may in part be driven by differences in their baseline and postvaccination influenza virus antibody repertoires. To this end, we characterized the anti-influenza virus IgG and IgA antibody repertoires using a broad set of influenza virus antigens, considering both conformational epitopes on whole viruses and recombinant HA proteins and linear epitopes using overlapping peptides spanning the HA and NA proteins of the Cal09 strain. We found striking differences in the antibody repertoires of HW and obese participants, despite the extensive heterogeneity of the baseline immune history profiles of individuals in both groups. BIH differences were primarily observed for the A/H1N1 influenza virus subtype, with individuals with obesity exhibiting lower IgG magnitudes to whole viruses and HA proteins, together with higher IgG magnitudes to Cal09 HA and NA peptides at baseline. At baseline, the obese group also displayed lower IgA magnitude to A/H1N1 HA proteins and narrower IgA breadth to Cal09 HA and NA peptides.

Consistent with previous studies (10), we demonstrate that individuals with obesity do respond to influenza vaccination. We then demonstrated that ranking individuals using their IgA and IgG BIH profiles was associated with both BMI and age: individuals with obesity were more likely to belong to the low-BIH group and elderly participants were more likely to belong to the high-BIH group when ranked by their IgG BIH to conformational epitopes. While the increased magnitude and breadth of responses to conformational epitopes in the elderly group may be driven by repeated exposures to influenza viruses as a result of vaccines and infections, the underlying causes of reduced magnitude and breadth in individuals with obesity are currently unknown. Concurrently, individuals with obesity were more likely to belong to the high-BIH group and elderly individuals were more likely to belong to the low-BIH group when ranked according to their IgG BIH to linear epitopes. The comparison of the antibody repertoire against linear and conformational A/H1N1 antigens in HW and obese participants demonstrated reduced ability in adults and individuals with obesity to develop an IgG response to whole virus or HA protein of the pandemic Cal09 strain, which may be associated with a biased IgG repertoire toward linear epitopes. In contrast, the A/H1N1 IgA repertoire of obese participants was significantly lower against both conformational and linear epitopes. Taken together, these findings suggest that the IgG and IgA repertoires develop independently or may be a function of infection during a novel pandemic. It is unclear why obesity and aging affect the IgG repertoire differently than the IgA repertoire, and additional mechanistic studies are required to address this question. The increased IgG binding to linear epitopes and reduced IgG binding to H1 proteins in the obese group could be due to a reduced capacity for affinity maturation, as affinity maturation often increases the number of interactions, necessitating binding to conformational epitopes (25).

Our analysis of the peptide antibody binding repertoire found that antibodies of obese and HW individuals target functionally distinct sites on the HA protein. In particular, antibodies from HW individuals preferentially targeted the HA head region, while obese individuals generated stronger responses to the HA stalk region, which is the target of broadly neutralizing antibodies (bNAbs). In our previous work, we showed that obese and HW mice vaccinated with an H7N9 influenza virus vaccine generated stalk antibody responses (26), but responses in obese mice were significantly lower than those in HW mice. To address the question of whether obese individuals may generate more broadly neutralizing stalk antibodies, we extracted the set of peptides that include the epitopes of 4 stalk bNAbs (see Materials and Methods) and compared the baseline and postvaccination magnitudes of responses in the obese and HW participants. We found that obese individuals generated significantly higher responses to these regions both at baseline and postvaccination (Fig. S12). While the exact reason for the difference between obese and HW antibody responses to influenza remains to be studied, childhood obesity and even maternal obesity are associated with increased risk of obesity in adulthood (27–30). These early obesity factors could lead to aberrant immune responses to vaccination and/or infection, leading to differential “immune imprinting” (31–35) compared to HW children or adults. Indeed, younger individuals with obesity in this cohort were more frequent in the low-BIH group for A/H1N1 whole viruses and HA proteins, which could bias responses later in life, especially if these individuals remain obese. The fact that the majority of aberrant responses were observed with the relatively “novel” antigen, the 2009 pandemic strain, indicates that primary or early responses to specific antigens can result in significantly skewed responses. Previous work has shown that obesity-associated changes in T-cell metabolism are associated with impairment of the T-cell response to influenza virus infection, which is not reversed with weight loss (36). These findings may also be important for responses to SARS-CoV-2 vaccines, which may be similarly skewed by responses to seasonal coronaviruses. Further studies on cohorts longitudinally sampled from birth to adulthood are needed to determine if childhood obesity or other factors may help to predict vaccination failure later in adulthood, even in HW individuals.

We found an association between low BIH to conformational epitopes and reduced protective anti-influenza virus antibody responses to the Cal09 strain, as measured by neutralizing antibodies and HAI titers. We also found a high prevalence of individuals with obesity in the low-BIH group. Finally, we showed that most of the individuals in the low-BIH group remain in this group postvaccination. Taken together, our data suggest that the majority of individuals with obesity have suboptimal baseline anti-influenza virus antibody repertoires, which remain suboptimal postvaccination. This may explain in part the reduced vaccine efficacy within the obese population (37). Interestingly, there were no significant differences between the baseline or postvaccination MN and HAI titers of the obese and HW groups within this study, highlighting the importance of ranking individuals by baseline immune history. These data also suggest that not all individuals with obesity fail to generate protective immune responses to influenza virus infection, as demonstrated by the existence of individuals with obesity in the high-BIH groups. Additional studies are required to further identify and study the differences between individuals with obesity within these two baseline groups.

One limitation of our study was that our study population was not controlled for several potential confounders. While age did not appear to differ significantly between HW and obese participants (Table 2), there were potential asymmetries for gender and race. In addition, other health factors potentially associated with obesity, such as diabetes, smoking history, and hypertension, were not accounted for and could potentially have skewed results. For example, 45% of obese participants and 7% of healthy-weight participants in our cohort suffered from type 2 diabetes. These factors could impact the specificity of our findings for obesity but would not diminish their potential relevance or vaccine strategies in an immunologically diverse population.

**TABLE 2:** Demographics of study participants

Group	*n*	Range (median)		No. of M/no. of F^*a*^	Races (%)
		BMI (kg/m^2^)	Age (yrs)		
Obese	105	30–46.9 (34.6)	20–82.6 (54.2)	42/63	Caucasian (55), African American (42), Hispanic (2), Asian (1)
Healthy wt	100	19–24.9 (22.7)	19–87 (57.8)	41/59	Caucasian (75), African American (16), Hispanic (2), Asian (6)

a M, males; F, females.

In addition to accumulated differences between the obese and HW groups in protection against seasonal human influenza virus strains, and possibly in responses to vaccines against future emergence of avian influenza pandemics, this work also has direct and timely relevance to the response to the current global COVID-19 pandemic. Due to the recent data on cocirculation of SARS-CoV-2 and influenza viruses in the Southern hemisphere during the 2022 winter months (38, 39) and the similarity of symptoms between disease manifestations, it is imperative for broad coverage of influenza vaccination to reduce clinical and diagnostic burden, as well as morbidity and mortality from influenza virus in an already strained health care system. With the growing rates of SARS-CoV-2 infections and vaccinations, and their combination, and given that obesity is also a risk factor for severe SARS-CoV-2 infection (5, 6), our study also highlights the need to study the anti-SARS-CoV-2 repertoires of obese and HW individuals.

Since individuals with obesity were reported as being more sensitive to Cal09 infection (40), our results suggest that an effective antibody response to Cal09 is associated with IgG antibodies to conformational epitopes. Additional functional studies are necessary to understand how to harness differential BIH and vaccine-induced immune responses of the obese population in order to induce more effective antibody responses in this population, especially once it is biased toward linear epitopes for IgG. Our data suggest that different influenza vaccines may be required for achieving optimal protection in an obese population. Overall, the design and development of different vaccines for individuals with obesity may need to be seriously taken into consideration, and clinical trials should take potential differences in BIH and vaccine-induced responses into account when choosing a representative “healthy” study population.

## MATERIALS AND METHODS

### Clinical data sets.

Participants were recruited as a part of a prospective observational study (41) carried out at the University of North Carolina at Chapel Hill Family Medicine Center, an academic outpatient primary care facility in Chapel Hill, NC. Recruitment criteria for this study included adults 18 years of age and older receiving the seasonal trivalent inactivated influenza vaccine (TIV) for the years 2010 and 2011 that included the following strains: A/H1N1/California/7/2009 (Cal09), A/H3N2/Perth/16/2009 (Perth09), and B/Brisbane/60/08 (Brisbane08). Exclusion criteria included immunosuppression, immunomodulatory or immunosuppressive drugs, acute febrile illness, history of hypersensitivity to any influenza vaccine components, history of Guillain-Barré syndrome, and use of theophylline preparations or warfarin. The study cohort included both obese (body mass index greater than 30 kg/m^2^; *n* = 105) and healthy-weight (18.5 ≤ BMI ≤ 24.9; *n* = 100) individuals. Patients recruited to the study provided a blood sample prior to vaccination (baseline; day 0) and 1 month (28 to 35 days) postvaccination. Blood was collected via antecubital puncture. Sera were collected using nonheparinized 10-mL Vacutainers and were allowed to clot at room temperature for 2 h before being separated by centrifugation at 800 × *g* for 10 min. Sera were then frozen at −80°C for subsequent analysis. All procedures were approved by the Biomedical Institutional Review Board at the University of North Carolina at Chapel Hill.

### Normalization of serum concentration.

Since the concentrations of different serum samples of the same individual may be different at different time points for a variety of reasons, serum concentrations were normalized by measuring the total protein concentration in the serum using Nanodrop and diluting all the samples to the same total protein concentration (10 mg/mL) with phosphate-buffered saline (PBS). These diluted samples were considered diluted 1:10, and additional dilutions were performed from them.

### Antigens.

β-Propiolactone (BPL)-inactivated whole influenza viruses were obtained from two sources. (i) Influenza viruses were grown in house in embryonated chicken eggs, BPL inactivated, and purified on sucrose columns as previously described (42), and their concentrations were determined by the hemagglutination assay, as previously described (42). (ii) Additional influenza viruses were obtained from the WHO (produced by the National Institute for Biological Standards and Control [NIBSC]) as reagents for a single radial diffusion (SRD) influenza virus potency assay with a known concentration. Recombinant HA proteins were purchased from Sino Biological (China) as purified His-tagged proteins. Most of them were produced in human HEK293 cell cultures, and some were produced in baculovirus-insect cells. Synthetic 20-aa peptides were synthesized at >90% purity by CPC Scientific (California, USA). Each peptide included an N-terminal KK tag as an amine group source for binding to the coated slides.

### Antigen microarray design and spotting.

To study the anti-influenza virus antibody repertoire, we designed and spotted two types of antigen microarrays. (i) An influenza virus protein (VP) microarray was spotted with a panel of 34 whole inactivated influenza viruses and 23 recombinant HA proteins (Table S1) that were selected to represent the antigenic diversity of 26 vaccine and 34 historical human influenza strains of the A/H1N1 and A/H3N2 subtypes and the B type from 1918 to 2016. Whole inactivated viruses were diluted in 0.0025% Triton X-100 and spotted at 2 hemagglutinating units (HAU)/μL for viruses that were grown in house in embryonated eggs or 4 μg HA/mL for WHO viruses. Recombinant HA proteins were diluted in 0.005% Triton X-100 and spotted at 16.25 μg/mL. (ii) A peptide microarray was spotted with 205 partially overlapping 20-aa peptides (with a 15-aa overlap) spanning the full-length HA (H1) and NA (N1) proteins of the pH1N1 2009 California vaccine strain (Cal09), which was included in the TIV vaccine tested in this study. Peptides were dissolved in 20 to 60% dimethyl sulfoxide (DMSO) to a 2-mg/mL solution, depending on the peptide hydrophobicity, and spotted at 1 mg/mL in 0.0025% Triton X-100. Microarrays were spotted using a Scienion Sx spotter (Scienion, Germany) on Hydrogel-coated slides that covalently bind amine groups (Nexterion Slide H; Schott, Germany). VP and peptide slides were printed separately, and all slides used for profiling the whole cohort were printed in a single batch to avoid potential batch effects. Each antigen was spotted in triplicate.

### Hybridization of antigen microarrays.

Human serum samples were diluted 1:3,000 for measuring IgG and 1:300 for measuring IgA in an incubation buffer that contained 1% bovine serum albumin (BSA) in 0.025% PBST (0.025% Tween 20 in PBS). The spotted slides were blocked by 1 h incubation on a rocker at room temperature (RT) with a chemical blocking solution (50 mM ethanolamine, 50 mM borate [pH 9.0]). After blocking, the slides were washed twice with 0.05% PBST, twice with PBS, and once with double-distilled water (DDW) (each wash, 3 min on the rocker) and dried by centrifugation at RT for 5 min at 800 × *g*. Then the microarrays were hybridized with the diluted serum samples in divided trays (PepperPrint) for 2 h at RT. Following washings as described above, the microarrays were incubated for 45 min with Alexa Fluor 647-labeled polyclonal anti-human IgG antibody at a 1:1,000 dilution (Jackson ImmunoResearch catalog no. 709-605-149) or Alexa Fluor 488-conjugated polyclonal anti-human IgA antibody at a 1:6,000 dilution (Jackson ImmunoResearch catalog no. 109-545-011). The secondary antibodies were diluted in 1% BSA in 0.025% PBST. To detect bound antibodies, slides were scanned on a two-laser GenePix 4400A scanner (Molecular Devices). In each run, several arrays were incubated with an incubation buffer only, without sample, to measure the background staining of each spot. Due to technical problems (high background or limited volume of some of the sera), a small number of the samples were not hybridized or analyzed. The analysis presented here included serum samples from 104 obese and 99 HW individuals for peptide arrays, from 94 obese and 95 HW individuals for IgG VP arrays, and from 87 obese and 87 HW individuals for IgA VP arrays.

### Analysis of microarray results.

Scanned slides were annotated using GenePix Pro version 7 (Molecular Devices) to obtain the mean fluorescence intensity (0 ≤ MFI ≤ 65,000) of each spot. The local background fluorescence intensity was subtracted from each spot’s MFI. We also subtracted the MFI values of an array incubated with BSA from the MFI values of all samples analyzed. The background median MFI−B staining of each antigen, as measured by microarrays incubated with an incubation buffer only, was subtracted from each median MFI−B result. Data analysis was conducted using an in-house pipeline written in Python. The vaccine-induced change in the antibody level to a given antigen was calculated as the fold change rise from the baseline level, or as a baseline-subtracted response.

### Magnitude and breadth summary statistics.

Normalized microarray results were analyzed using Python scripts. For a given serum sample and a set of antigens, the magnitude and breadth were defined as follows: “magnitude” denotes the sum of median MFI−B results in response to a given set of antigens, and “breadth” denotes the number of antigens in a given set with a median MFI−B higher than a selected threshold. Thresholds were defined manually and related to background responses observed for each antigen type. Specifically, the following thresholds were used: an MFI of >2,000 for peptides and recombinant HA proteins, and an MFI of >4,000 for whole viruses. Magnitude and breadth were calculated for each participant at each time point and also for the postvaccination baseline-subtracted responses. We computed magnitude and breadth to the following sets of antigens: Cal09 H1 peptides, Cal09 N1 peptides, subtype B HA proteins, subtype A/H1N1 HA proteins, subtype A/H3N2 HA proteins, subtype B whole viruses, subtype A/H1N1 whole viruses, and subtype A/H3N2 whole viruses.

### BIH ranking.

All participants with successful microarray results were ranked according to the baseline magnitude or breadth to A/H1N1 whole viruses or HA proteins of A/H1N1 strains (H1) or to Cal09 H1 peptides or Cal09 N1 peptides. For each ranking, the participants were divided into quartiles, and the two extreme quartiles of highest and lowest scores, termed high BIH and low BIH, respectively, were compared. The number of individuals in each group varied by assay due to technical issues such as sample unavailability and high background (Table 3).

**TABLE 3:** Numbers of samples

Antigens	No. of samples			
	Low BIH	Med BIH	High BIH	Total
Viruses and proteins, IgG	47	95	47	189
Viruses and proteins, IgA	44	86	44	174
H1 and N1 peptides, IgG	51	101	51	203
H1 and N1 peptides, IgA	51	101	51	203

### Vaccine responder ranking.

For each individual, we computed the baseline-adjusted response to the vaccine for each peptide in the peptide microarray, by subtracting the baseline binding of antibodies to this peptide from the postvaccination antibody binding. All participants with peptide microarray results (*n* = 205) were ranked according to the magnitude or breadth of baseline-adjusted responses to Cal09 H1 or N1 peptides. The participants were divided into quartiles, and the quartiles with the highest and lowest baseline-adjusted responses, termed high responders and low responders, respectively, were compared.

### Statistical analysis.

We used 2-sided hypothesis tests (Wilcoxon rank-sum and Fisher’s exact tests) to test for differences between the distribution of breadth and magnitude scores defined above. Differences between baseline and postvaccination responses within each group were tested using the Wilcoxon signed-rank test. The RR and 95% CI for individuals with obesity or individuals <65 years old to be in the lowest quartile of IgG or IgA BIH magnitude to whole viruses, recombinant proteins, H1 peptides, or N1 peptides, compared to healthy-weight individuals or individuals >65 years old, was calculated using the Python statsmodels package and an on-line calculator from MedCalk (https://www.medcalc.org/calc/relative_risk.php). When the 95% CI includes 1, then being obese or <65 years old may have no effect on the participant being in the lowest quartile of BIH response.

To predict the group of participants based on the immune-history profiles, we used a logistic regression model, and a generalized linear model (GLM) was used to predict the vaccine-induced immune responses using a logistic model for binary variables and a linear model for continuous variables. All models were trained using leave-one-out cross validation. Predicted values were collected over all folds for computing the area under the curve (AUC) summary statistic. All continuous variables were standardized prior to training. Regularization was implemented using the elastic net package with different alpha and L1 weight parameters for each model. All models were trained using the Python statsmodels package.

### Spider plots.

Spider plots depicting the IgG or IgA antibody binding to HA proteins or whole-virus antigens in individual samples simply present the normalized median MFI−B result of each antigen after background subtraction (see “Analysis of microarray results” above). Normalization was performed for each antigen across all samples, such that the maximal median MFI−B result of this antigen across all baseline and postvaccination samples was set as 1, and all other binding results for this antigen in other samples were relative. In addition, the average of normalized IgG or IgA antibody binding results was calculated for each BIH quartile at each time point.

### Human IgG and IgA ELISA quantification.

Commercial ELISA kits (Bethyl Laboratories, USA; catalog no. E80-104 for human IgG and catalog no. E80-1026 for human IgA) were used to quantify the total human IgG and IgA concentrations in the normalized sera, using the manufacturer's instructions with the following modifications. The normalized serum samples were diluted 1:24,375 for human IgA ELISA and 1:243,750 for human IgG ELISA. The ELISAs were performed in 384-well white MaxiSorp Nunc plates (catalog no. 460372). The wells were coated with 17 μL/well of ELISA coating antibody in the coating buffer. Blocking and all washes were performed using 100 μL/well, and 30 μL/well diluted sera and standards were added in triplicate. Following washes, 30 μL/well horseradish peroxidase (HRP)-conjugated detection antibody was also added. Instead of TMB (3,3′,5,5′-tetramethylbenzidine), we used 30 μL/well of SuperSignal West Pico Plus chemiluminescent substrate (Thermo Scientific catalog no. 34579; the two reagents were mixed in a 1:1 ratio immediately before being added to the plate). Plates were scanned using a standard luminometer (Tecan Infinite M200 Pro) at 600 nm. The average of each triplicate was calculated, and total antibody concentration was concluded from the standard curve.

### Functional antibody assays (immunogenicity).

Cal09 microneutralization (MN) titers at baseline and postvaccination time points, as well as postvaccination hemagglutination inhibition (HAI) titers, were measured for only 76 of the individuals. HAI titer was determined in a blind fashion in accordance with World Health Organization guidelines for A/California/04/2009 wild-type virus. Luminescent MN assays were performed in a blind fashion as previously described using a reverse genetics A/California/04/2009 (pdmH1N1) virus containing NanoLuc luciferase (NLuc) on its polymerase segment 19 (43, 44).

### Scoring HA residues based using a logistic regression model.

A logistic regression model was trained based on H1/N1 peptide array results to predict obesity status. The model assigns weights to individual features (peptides) from both the HA and NA proteins of the Cal09 vaccine strain. We used the weights from the baseline IgA or the baseline IgG models. The weight of HA residue was defined by the maximal weight of peptides containing the given residue. Specifically, HA residues with high positive weights (>5% of maximal weight) were associated with the obese group, and HA residues with high negative weights (>5% of maximal weight) were associated with the HW group.

### Curating HA domains and sites.

Residues comprising the HA domain could be mapped to the following sites based on a previous publication (45). The numbering here is according to PDB ID 3LZG (46). HA1 chain A occupies positions 11 to 325, HA2 chain B occupies positions 1 to 175, fusion domain chain A occupies positions 11 to 64 and 276 to 324, and fusion domain chain B occupies positions 1 to 160. RBS chain A occupies positions 115 to 264, and esterase domain chain A occupies positions 66 to 116 and 166 to 277. For conserved HA glycosylation sites, chain A occupies positions 20 to 23, 33 to 35, and 289 to 291, and chain B occupies positions 154 to 156. Cal09 glycosylation site chain A occupies positions 278 to 280. For antigenic sites (all residues comprising Cb, Sb, Sa, Ca1, and Ca2), Cb antigenic site chain A occupies positions 79 to 84; Sb antigenic site chain A occupies positions 187 to 199; Sa antigenic site chain A occupies positions 128, 129, 156 to 161, and 162 to 168; Ca1 antigenic site chain A occupies positions 168 to 174, 206 to 209, and 238 to 241; and Ca2 antigenic site chain A occupies positions 139 to 145 and 224 to 226.

### Statistical significance test for enrichment of scored residues in functional domains.

A permutation test was used to examine the significance of enrichment of residues selected by the logistic regression model in each domain. We computed the difference between the percentage of positively scored residues and negatively scored residues within each domain. (Indicating obese and HW status, respectively), we use *N* to denote the number of residues in the HA protein (*n* = 498), *S* for the number of residues making up a given domain, and *D* for the difference between weights of positive and negative residues within a given domain. The permutation test involved 10,000 iterations. In each iteration, we sampled *S* residues out of *N* and calculated *D*. *P* values were calculated by counting the number of iterations where *D*_permuted_ > *D*_original_ and dividing by the number of iterations.

### Curating stalk broadly neutralizing binding sites.

We utilized a set of 4 stalk broadly neutralizing antibodies with solved structures: C49114 (PDB IDs 5CJQ and 5CJS), CR626 (PDB ID 3GBN), F10 (PDB ID 3FKU), and FI6v3 (PDB ID 3ZTN). For each of these MAbs, epitope residues were calculated as HA residues at less than a 4-Å atomic distance from the antibody. We then extracted the set of stalk peptides that contained positions within the contact sites of these antibodies, excluding peptides with a single contact position. The final set of peptides included all 20-mer peptides starting at the following positions: 21, 31, 36, 41, 346, 351, 356, 361, 376, 381, 386, and 391 (47–49).

### Data availability.

All row data and code used in the analysis are available at the Hertz lab website: https://www.hertz-lab.org/.
