# Supplementary material for: Obesity Is Associated with an Impaired Baseline Repertoire of Anti-Influenza Virus Antibodies
Source: Microbiol Spectr. 2023 Apr 26;11(3):e00010-23. doi: 10.1128/spectrum.00010-23 (PMC10269616; doi:10.1128/spectrum.00010-23)
Supplement: Supplemental file 1 — Supplemental material. Download spectrum.00010-23-s0001.pdf, PDF file, 6.3 MB [file spectrum.00010-23-s0001.pdf]

Supplementary Figure 1

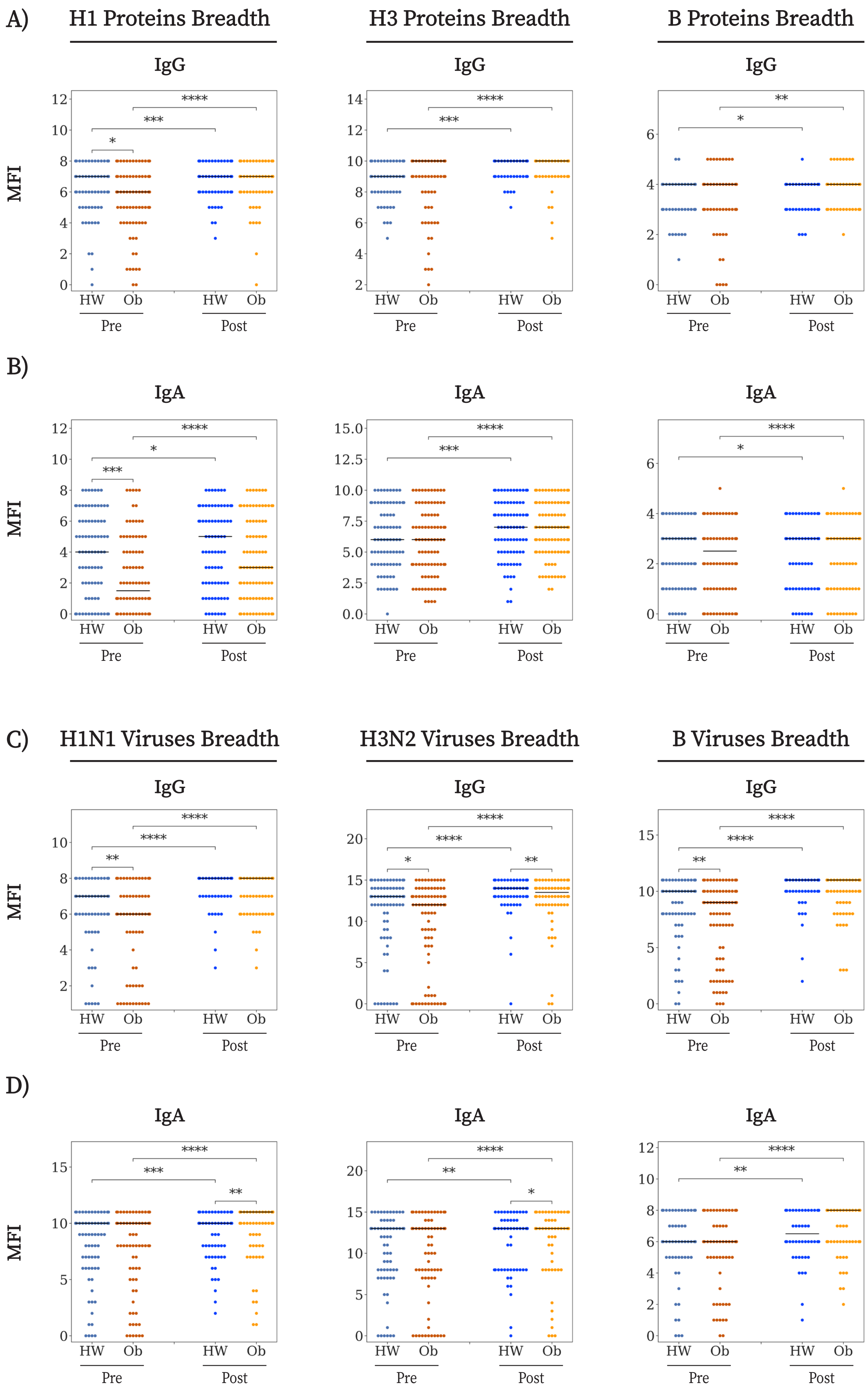

Fig. S1. Baseline immune history and post-vaccination responses to influenza H3N2 viruses and HA proteins. Baseline and post-vaccination antibody levels and fold-rise of 89 healthy-weight (HW) and 100 obese (Ob) individuals to a panel of 10 H3 proteins and 15 H3N2 BPL-inactivated viruses spotted on antigen microarrays (see Table S1). (**A**) IgG binding to the HA of H3N2 A/Perth/16/2009 (Perth09). (**B**) IgG magnitude to a panel of 10 H3 proteins. (**C**) IgA binding to the HA of Perth09. (**D**) IgA magnitude to a panel of 10 H3 proteins. **(E)** IgG binding to Perth09 BPL-inactivated virus. (**F**) IgG magnitude to a panel of 15 H3N2 viruses. (**G**) IgA binding to Perth09 BPL-inactivated virus. (**H**) IgA magnitude to a panel of 15 H3N2 viruses. Lines represent the median fluorescence intensity (MFI), the boxes denote the 25th and 75th percentiles, and the error bars represent 1.5 times the interquartile range. Statistical significance was assessed using the Wilcoxon signed rank test (baseline vs. post-vaccination) and the Wilcoxon rank-sum test (HW vs. obese). * $p<0.05$, ** $p<0.005$, *** $p<0.0005$.

Supplementary Figure 2

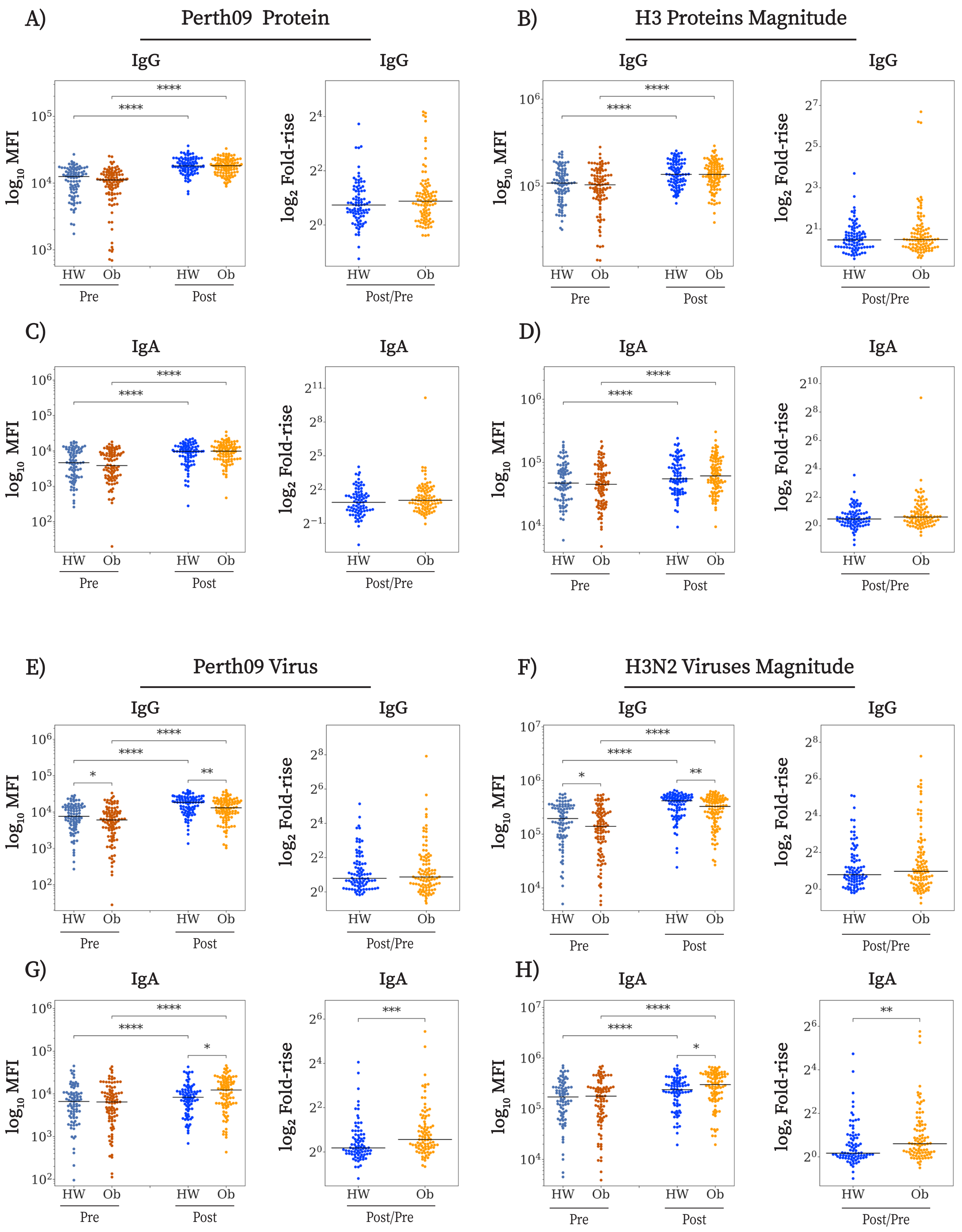

Fig. S2. Baseline immune history and post-vaccination responses to influenza B viruses and HA proteins. Baseline and post-vaccination antibody levels and fold-rise of 89 healthy-weight (HW) and 100 obese (Ob) individuals to a panel of 5 HA proteins and 10 BPL-inactivated viruses of influenza B strains spotted on antigen microarrays (see Table S1). (**A**) IgG binding to the HA of B/Brisbane/60/2008 (Brisbane08). (**B**) IgG magnitude to a panel of HA proteins of 5 influenza B strains. (**C**) IgA binding to the HA of Brisbane08. (**D**) IgA magnitude to a panel of HA proteins of 5 influenza B strains. **(E)** IgG binding to Brisbane08 BPL-inactivated virus. (**F**) IgG magnitude to a panel of 10 influenza B viruses. (**G**) IgA binding to Brisbane08 BPL-inactivated virus. (**H**) IgA magnitude to a panel of 10 influenza B viruses. Lines represent the median fluorescence intensity (MFI), the boxes denote the 25th and 75th percentiles, and the error bars represent 1.5 times the interquartile range. Statistical significance was assessed using the Wilcoxon signed rank test (baseline vs. post-vaccination) and the Wilcoxon rank-sum test (HW vs. obese). * $p<0.05$, ** $p<0.005$, *** $p<0.0005$.

Supplementary Figure 3

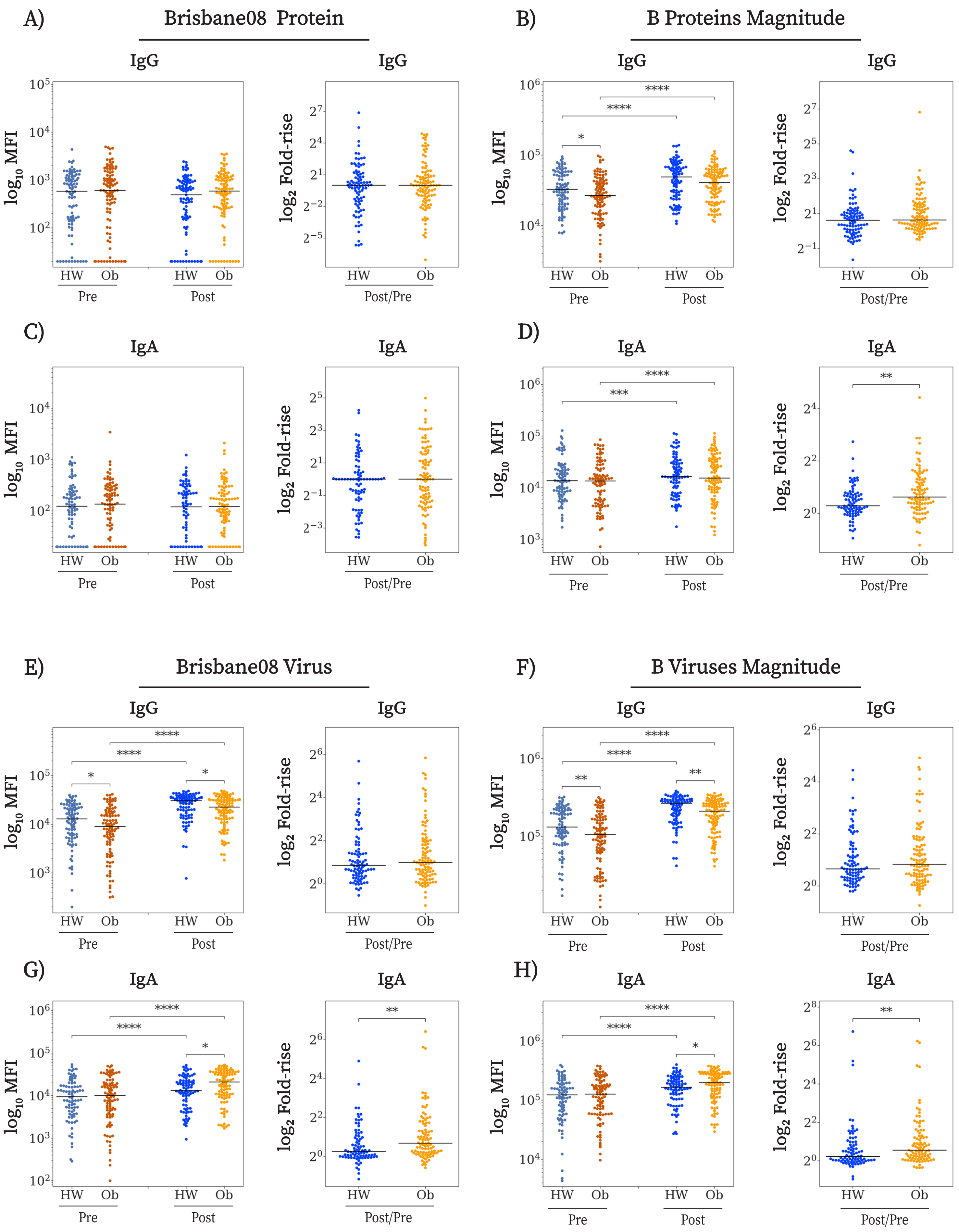

Fig. S3. Breadth of anti-influenza antibodies at baseline and post-vaccination. Baseline and post-vaccination antibody breadth of 89 healthy-weight (HW) and 100 obese (0B) individuals to a panel of 23 HA proteins (A-B) and 34 BPL-inactivated viruses (C-D) spotted on antigen microarrays (see Table S1). Breadth was defined by the number of antigens to which an individual responded to (see methods). (**A-B**) IgG (A) or IgA (B) breadth to a panel of 8 H1, 10 H3 and 5 B HA proteins. (**C-D**) IgG (C) or IgA (D) breadth to a panel of 11 H1N1, 15 H3N2 and 10 B strains of influenza viruses. Lines represent the median fluorescence intensity (MFI), the boxes denote the 25th and 75th percentiles, and the error bars represent 1.5 times the interquartile range. Statistical significance was assessed using the Wilcoxon signed rank test (baseline vs. post-vaccination) and the Wilcoxon rank-sum test (HW vs. obese). * $p<0.05$, ** $p<0.005$, *** $p<0.0005$.

Supplementary Figure 4

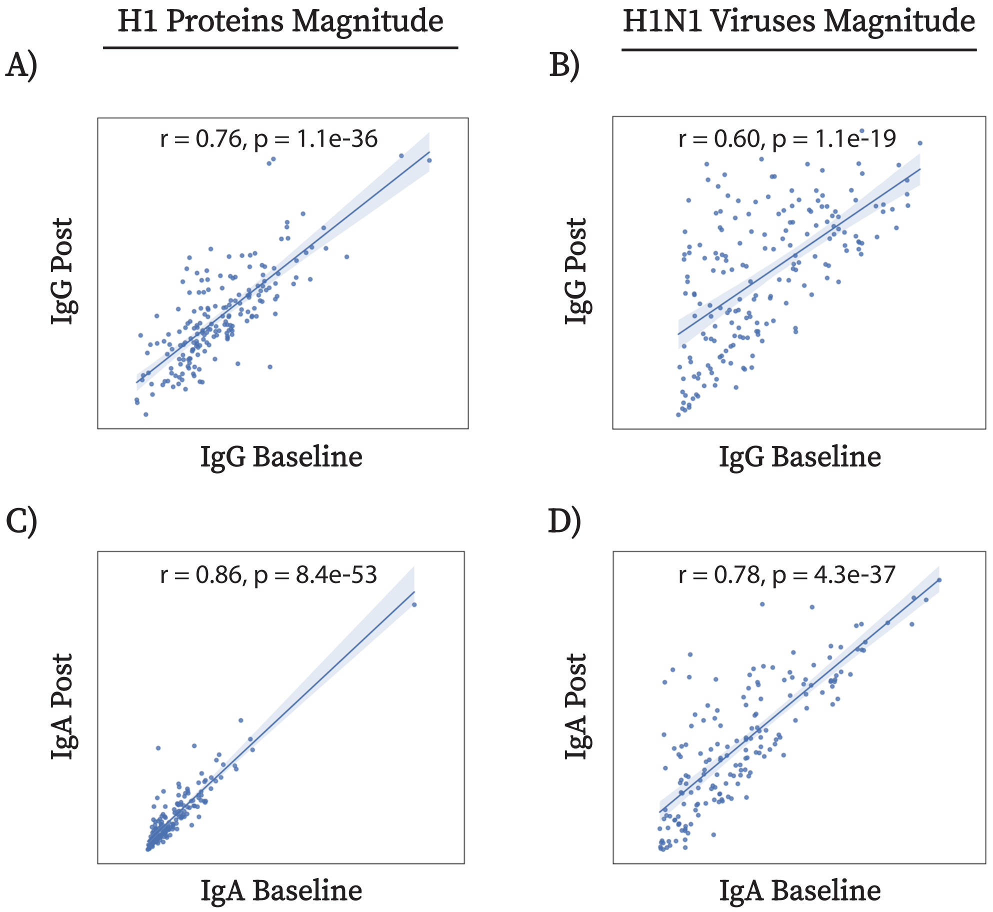

Fig. S4. Baseline and post-vaccination H1N1 antibody profiles are significantly correlated. Scatter plots of the baseline (x-axis) and post-vaccination (y-axis) magnitudes of antibody responses to H1N1 antigens. Each dot represents a single individual. Lines represent the estimated linear regression and shaded areas denote the confidence interval. Spearman correlation values are reported for each panel. (**A**) IgG magnitude of H1 proteins. (**B**) IgG magnitude of H1N1 viruses. (**C**) IgA magnitude of H1 proteins. (**D**) IgA magnitude of H1N1 viruses.

Supplementary Figure 5

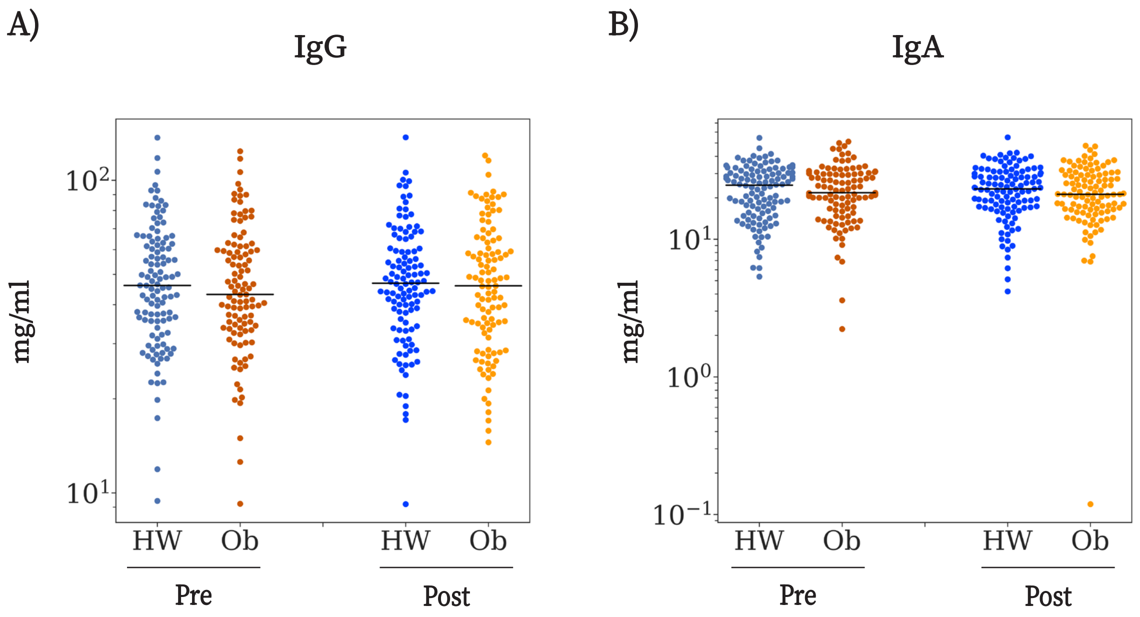

Fig. S5. Total IgG and IgA serum titers are not significantly different in obese and healthy-weight individuals. Cumulative distribution plots comparing the baseline and post-vaccination total level of IgG (**A**) and IgA (**B**) as measured using sandwich ELISA. Black lines represent the median titer.

Supplementary Figure 6

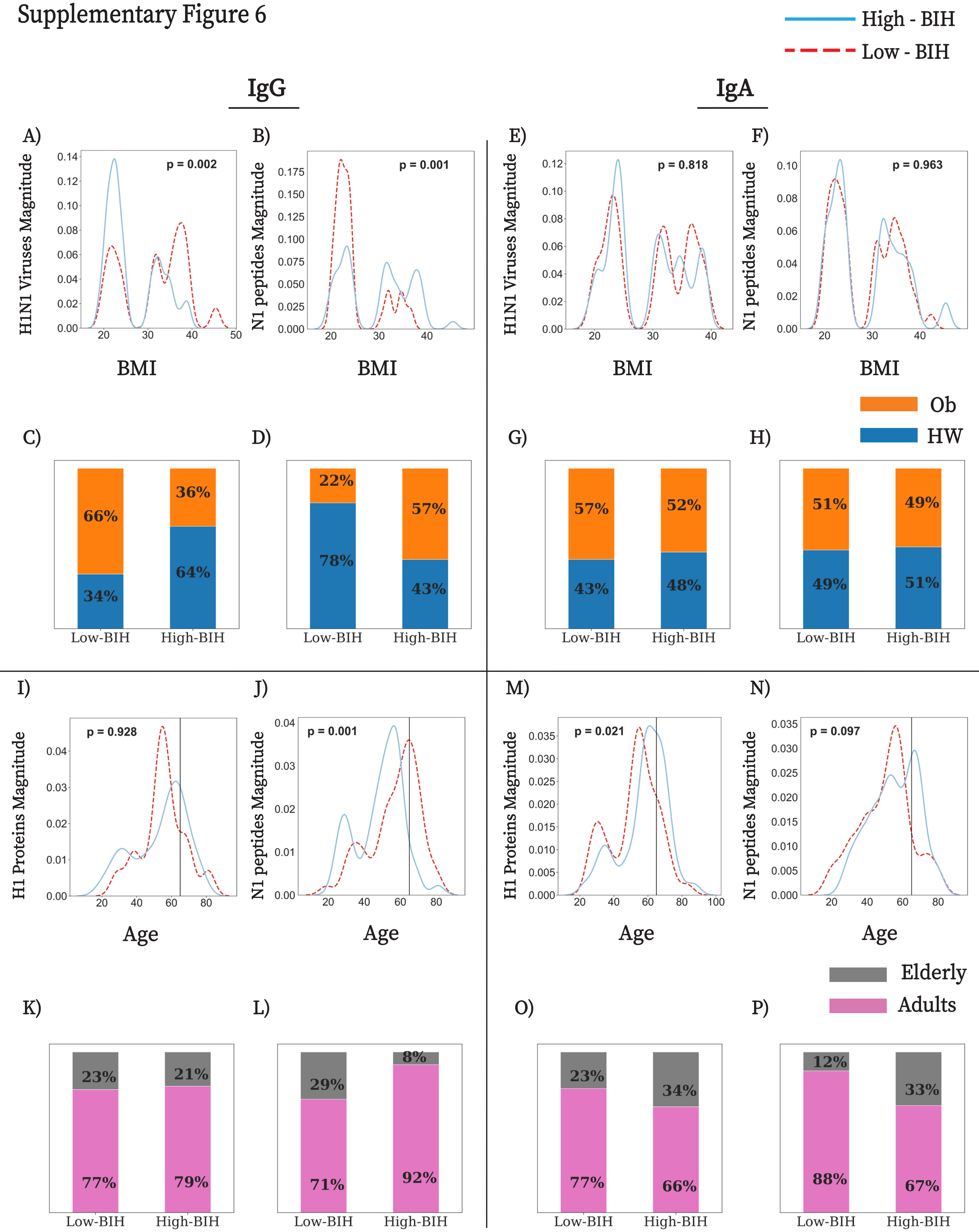

Fig. S6. BMI and age are associated with baseline-immune history to influenza H1N1. (**A-H**) The distributions of BMI in the low-BIH groups (dashed red line) and the high-BIH groups (solid blue line) ranked by (**A**) IgG Magnitude against H1N1 viruses; (**B**) IgG Magnitude to N1 peptides; (**C-D**) The percentage of obese (orange) and HW (blue) participants in the low- and high-BIH groups for (**C**) IgG Magnitude against H1N1 viruses; and (**D**) IgG Magnitude to N1 peptides; (**E**) IgA magnitude to H1N1 viruses; (**F**) IgA magnitude to N1 peptides; (**G-H**) The percentage of obese participants in the low- and high-BIH for (**G**) IgA Magnitude against H1N1 viruses; and (**H**) IgA Magnitude to N1 peptides. Overweight participants (25 < BMI < 30) were excluded from our analysis. (**I-P**) Distributions by age group comparing adult (<65) and elderly individuals within the low-BIH group and the high-BIH groups ranked by: (**I**) IgG Magnitude against H1 proteins; (**J**) IgG Magnitude to N1 peptides; (**K-L**) The percentages of elderly (>65 y, gray) individuals in the low- and high-BIH groups sorted by (**K**) IgG magnitude against H1N1 proteins; and (**L**) IgG magnitude to N1 peptides; (**M**) IgA magnitude to H1 proteins; (**N**) IgA magnitude to N1 peptides; (**O-P**) The percentages of elderly (>65 y, gray) individuals in the low- and high-BIH groups sorted by (**O**) IgA magnitude against H1N1 proteins; and (**P**) IgA magnitude to N1 peptides. P values comparing the differences between the BMI (A-H) or age (I-P) distributions of the low-BIH and high-BIH groups were determined using the Wilcoxon ranksum test. The number of participants in each low- or high-BIH group are listed in **Table S2**.

Supplementary Figure 7

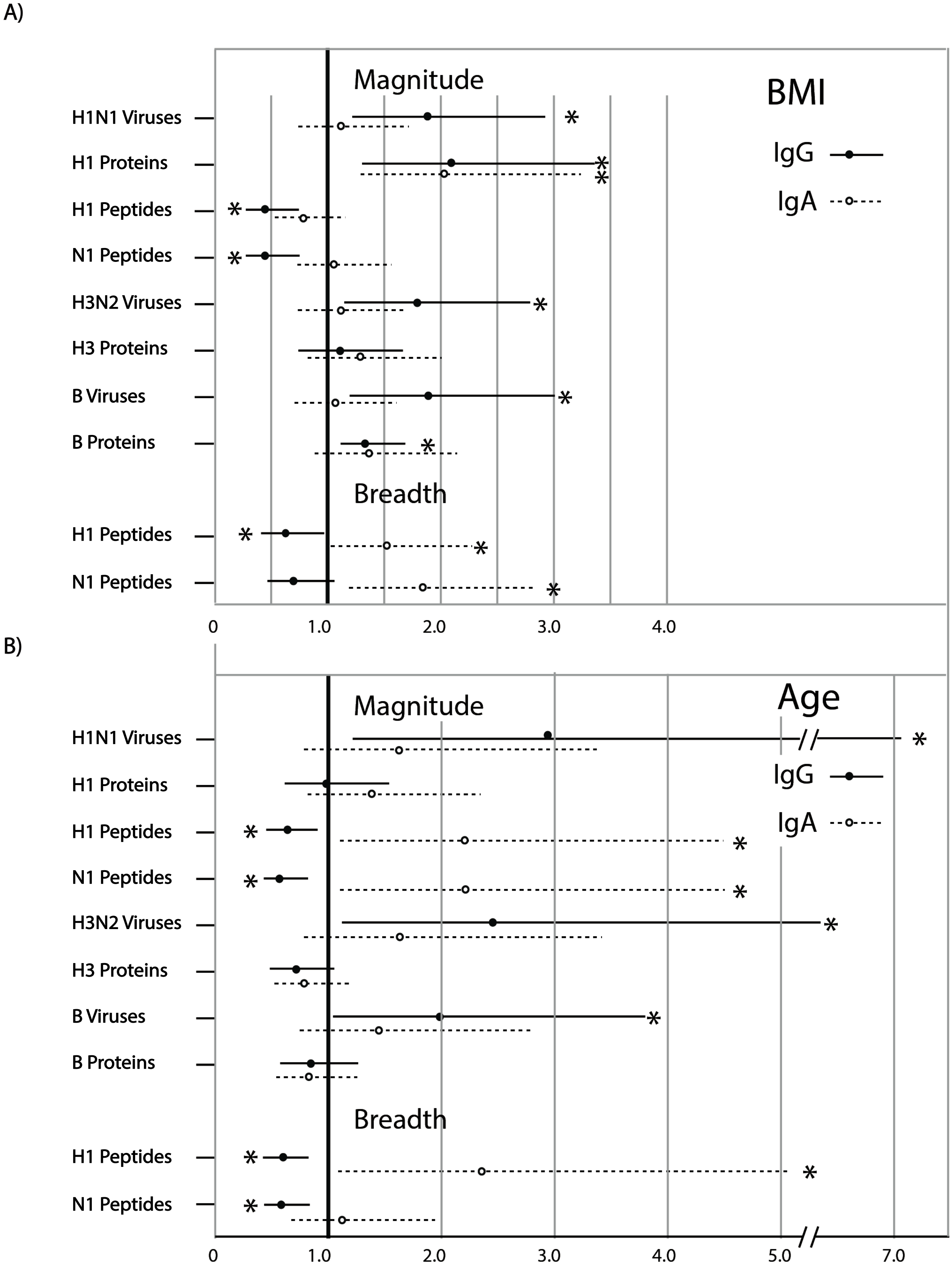

Fig. S7. Relative risk for being in the lowest quartile of magnitude or breadth of BIH-IgG responses. (A) The relative risk and 95% CI for obese individuals to belong to the low-BIH quartile as compared to healthy-weight individuals for IgG (black filled circles) and IgA (white filled circles). **(B)** Relative risk (RR) and 95% CI for adult individuals (<65 y) to belong to the low-BIH group as compared to elderly (>65) individuals for IgG (black filled circles) and IgA (white filled circles). Significant associations are marked by *.

Supplementary Figure 8

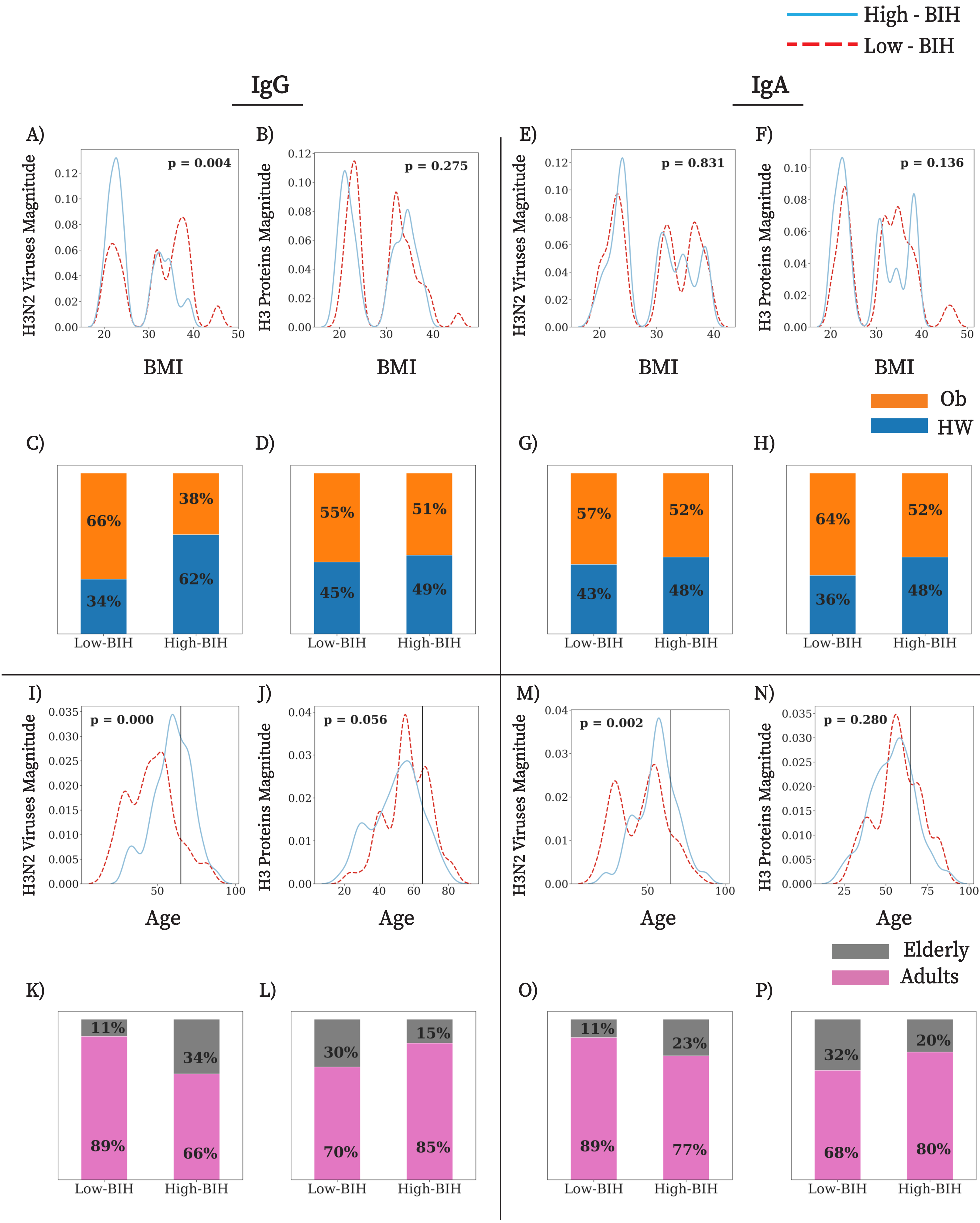

Fig. S8. BMI and age associations with influenza H3N2 baseline-immune history. (A-H) Obesity was associated with low IgG-BIH to H3N2 viruses. The distributions of BMI in the low-BIH group (dashed red line) and the high-BIH group (solid blue line) ranked by H3N2 antigens. Overweight participants (25 < BMI < 30) were excluded from our analysis. The participants BIH were ranked by: (**A**) IgG magnitude against H3N2 viruses; (**B**) IgG magnitude to H3N2 HA (H3) proteins; (**C-D**) The percentage of obese (orange) and HW (blue) participants in the low- and high-BIH groups for (**C**) IgG Magnitude against H3N2 viruses; and (**D**) IgG Magnitude to H3 proteins; (**E**) IgA magnitude to H3N2 viruses; (**F**) IgA magnitude to H3 proteins; (**G-H**) The percentage of obese participants in the low- and high-BIH for (**G**) IgA Magnitude against H3N2 viruses; and (**H**) IgA Magnitude to H3 proteins. (**I-P**) Old age (>65) was associated with high IgA and IgG BIH to H3N2 viruses. Age distributions within the low-BIH group and the high-BIH group ranked by H3N2 antigens: (**I**) IgG Magnitude against H3N2 viruses; (**J**) IgG Magnitude to H3 proteins; (**K-L**) The percentages of elderly (>65 y, gray) individuals in the low- and high-BIH groups sorted by (**K**) IgG magnitude against H3N2 viruses; and (**L**) IgG magnitude to H3 proteins; (**M**) IgA magnitude to H3N2 viruses; (**N**) IgA magnitude to H3 proteins; (**O-P**) The percentages of elderly (>65 y, gray) individuals in the low- and high-BIH groups sorted by (**O**) IgA magnitude against H3N2 viruses; and (**P**) IgA magnitude to H3 proteins. P values for differences between the BMI (A-H) or age (I-P) distributions of the low-BIH and high-BIH groups were determined using the Wilcoxon ranksum test.

Supplementary Figure 9

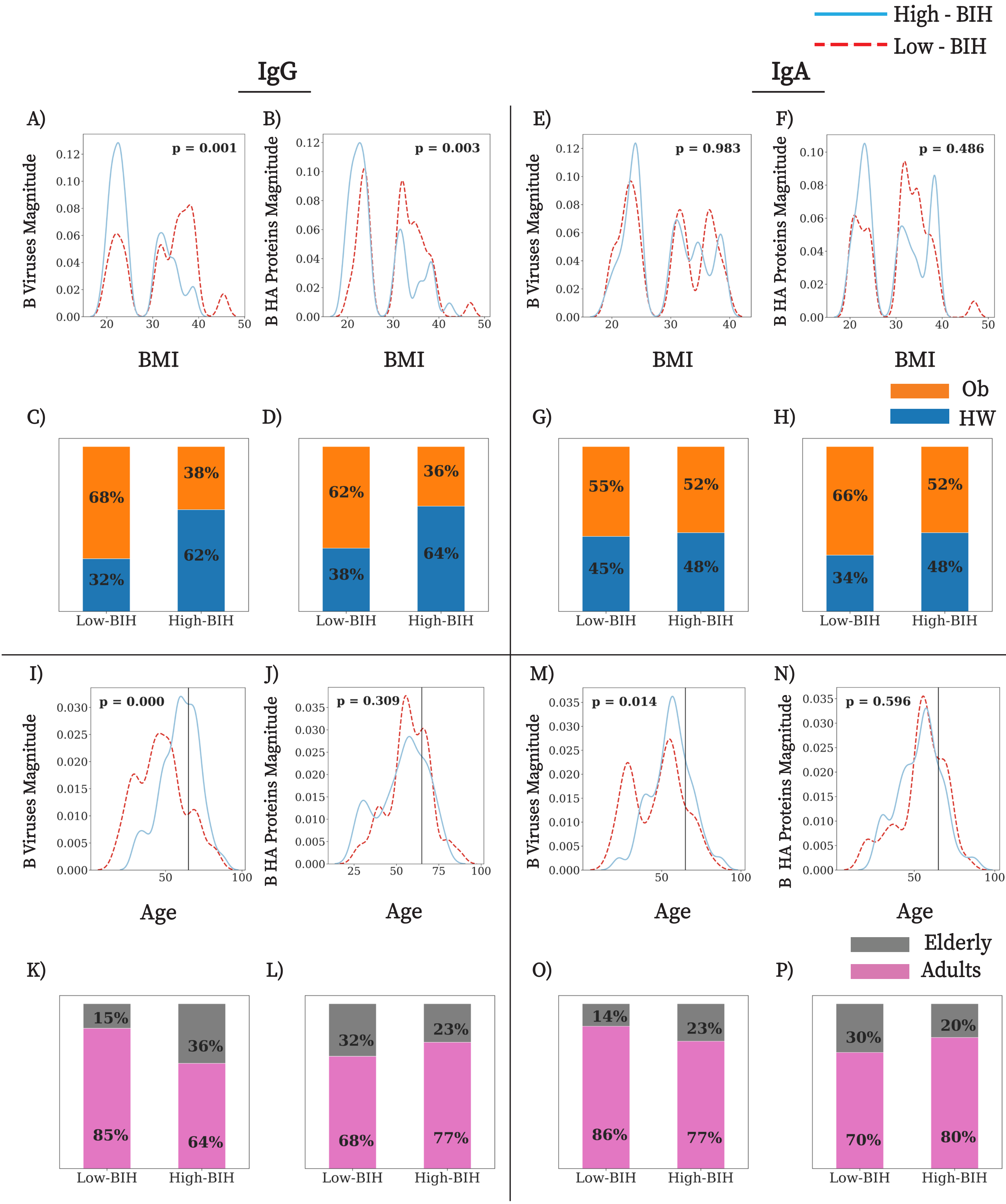

Fig. S9. BMI and age associations with influenza B baseline-immune history. (**A-H**) Obesity was associated with low IgG-BIH to B viruses and B HA proteins. The distributions of BMI in the low-BIH group (dashed red line) and the high-BIH group (solid blue line) ranked by B antigens. Overweight participants (25 < BMI < 30) were excluded from our analysis. The percentage of obese participants in the low-and high-BIH groups are listed. The participants BIH were ranked by: (**A**) IgG magnitude against B viruses; (**B**) IgG magnitude to B HA proteins; (**C-D**) The percentage of obese (orange) and HW (blue) participants in the low- and high-BIH groups for (**C**) IgG Magnitude against B viruses; and (**D**) IgG Magnitude to B HA proteins; (**E**) IgA magnitude to B viruses; (**F**) IgA magnitude to B HA proteins; (**G-H**) The percentage of obese participants in the low- and high-BIH for (**G**) IgA Magnitude against B viruses; and (**H**) IgA Magnitude to B HA proteins. (**I-P**) Old age (>65) was associated with high IgA and IgG BIH to B viruses. Age distributions within the low-BIH group and the high-BIH group ranked by B antigens: (**I**) IgG Magnitude against B viruses; (**J**) IgG Magnitude to B HA proteins; (**K-L**) The percentages of elderly (>65 y, gray) individuals in the low- and high-BIH groups sorted by (**K**) IgG magnitude against B viruses; and (**L**) IgG magnitude to B HA proteins; (**M**) IgA magnitude to B viruses; (**N**) IgA magnitude to B HA proteins; (**O-P**) The percentages of elderly (>65 y, gray) individuals in the low- and high-BIH groups sorted by (**O**) IgA magnitude against B viruses; and (**P**) IgA magnitude to B HA proteins. P values for differences between the BMI (A-H) or age (I-P) distributions of the low-BIH and high-BIH groups were determined using the Wilcoxon ranksum test.

Supplementary Figure 10

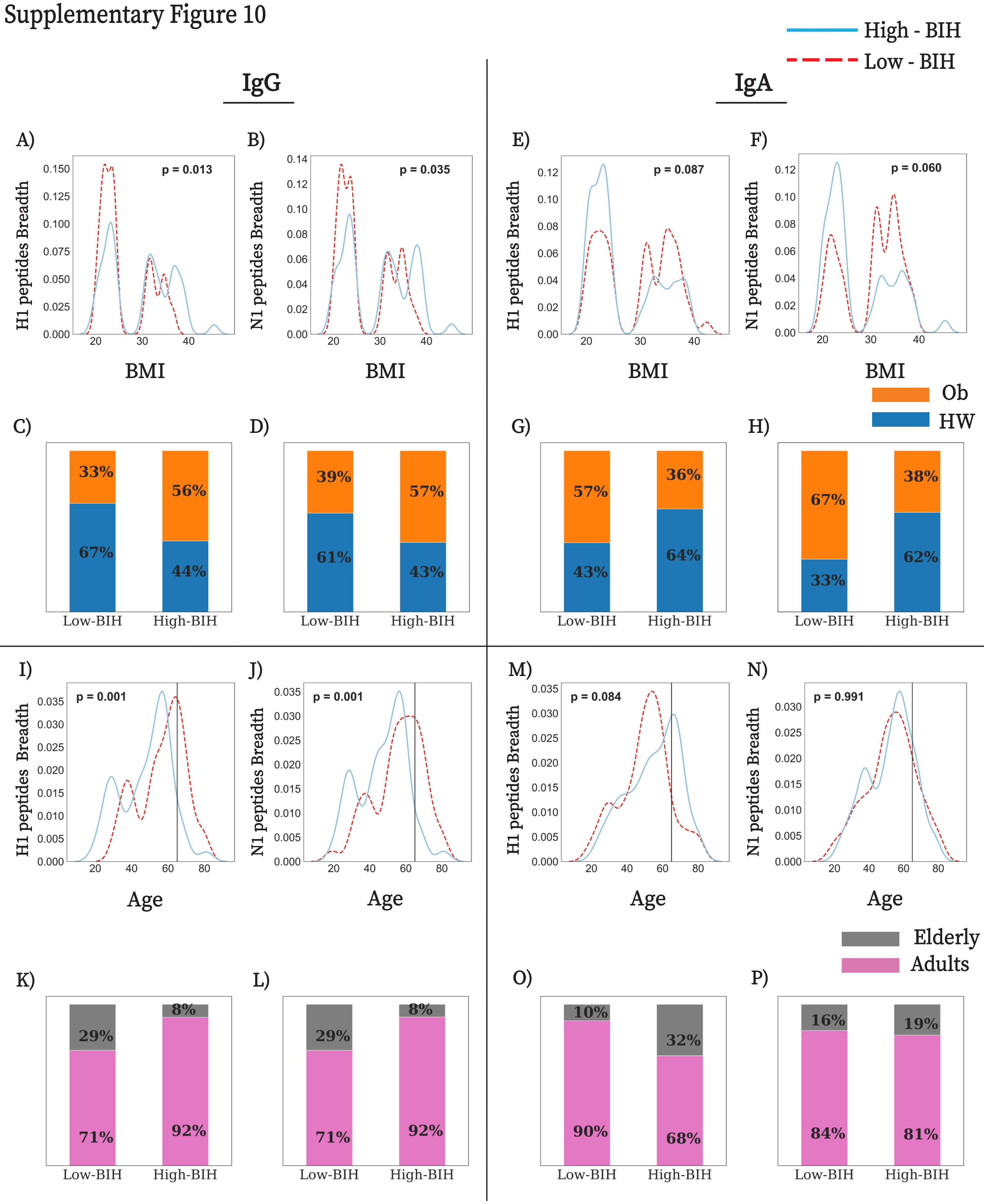

Fig. S10. BMI and age associations with baseline-immune history of antibody breadth. (**A-H**) The distributions of BMI in the low-BIH groups (dashed red line) and the high-BIH groups (solid blue line) ranked by (**A**) IgG breadth to H1 peptides; (**B**) IgG breadth to N1 peptides; (**C-D**) The percentage of obese (orange) and HW (blue) participants in the low- and high-BIH groups for (**C**) IgG breadth against H1 peptides; and (**D**) IgG breadth to N1 peptides; (**E**) IgA breadth to H1 peptides; (**F**) IgA breadth to N1 peptides; (**G-H**) The percentage of obese participants in the low- and high-BIH for (**G**) IgA breadth against H1 peptides; and (**H**) IgA breadth to N1 peptides. Overweight participants (25 < BMI < 30) were excluded from our analysis. (**I-P**) Distributions by age group comparing adult (<65) and elderly individuals within the low-BIH group and the high-BIH groups ranked by: (**I**) IgG breadth against H1 peptides; (**J**) IgG breadth to N1 peptides; (**K-L**) The percentages of elderly (>65 y, gray) individuals in the low- and high-BIH groups sorted by (**K**) IgG breadth against H1 peptides; and (**L**) IgG breadth to N1 peptides; (**M**) IgA breadth to H1 peptides; (**N**) IgA breadth to N1 peptides; (**O-P**) The percentages of elderly (>65 y, gray) individuals in the low- and high-BIH groups sorted by (**O**) IgA breadth against H1 peptides; and (**P**) IgA breadth to N1 peptides. P values comparing the differences between the BMI (A-H) or age (I-P) distributions of the low-BIH and high-BIH groups were determined using the Wilcoxon ranksum test. The number of participants in each low- or high-BIH group are listed in **Table S2**.

Supplementary Figure 11

A) IgG BIH HA1

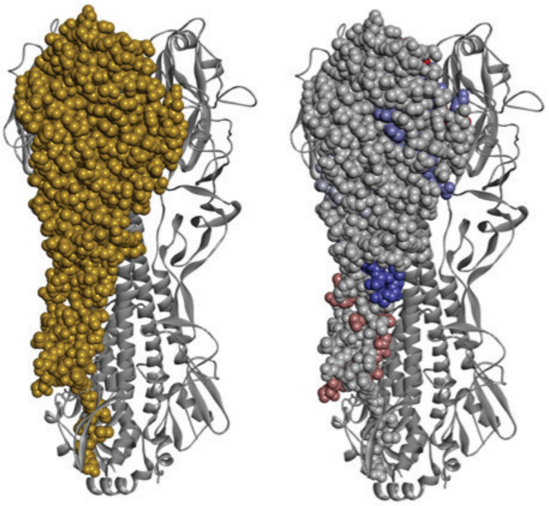

B) IgG BIH HA2

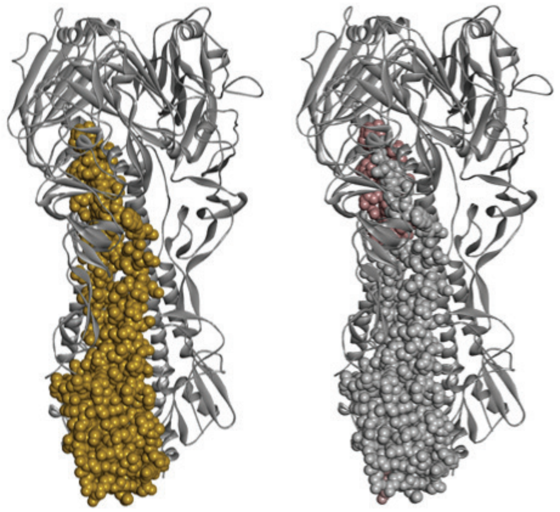

C) IgA BIH Fusion domain

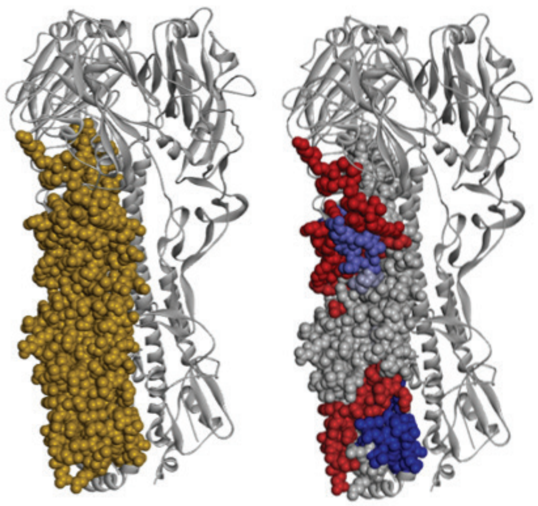

D) IgA BIH Receptor Binding Site

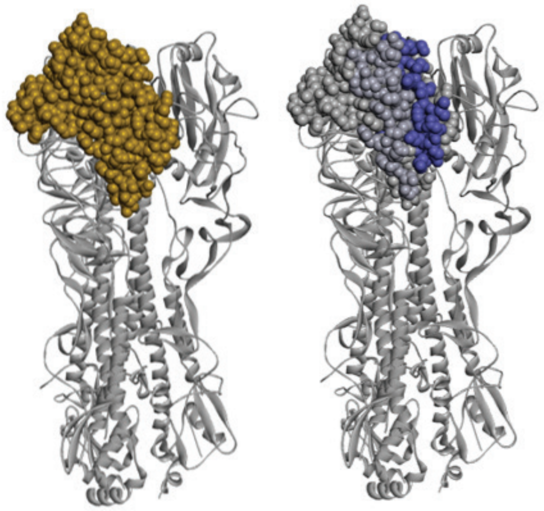

E) IgA BIH Esterase domain

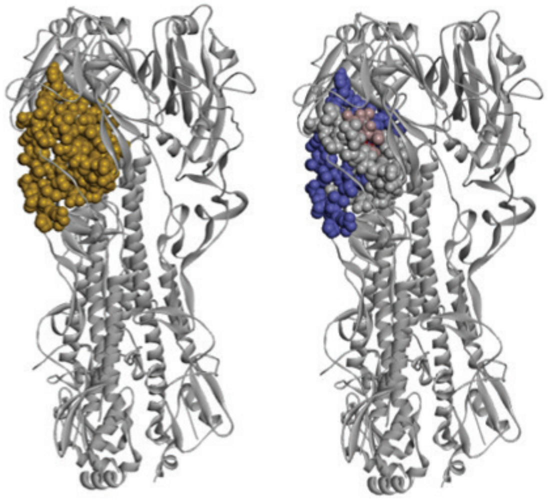

Fig. S11. Cal09 HA domains are differentially targeted by BIH IgG and IgA antibodies of obese and healthy weight participants. A logistic regression model was trained to discriminate between HW and obese individuals using the IgG and IgA antibody profiles to the HA peptides of the Cal09 vaccine strain. The weights assigned by the model were used to score individual amino acids on the HA protein based on the maximal weight of a given position across all of the peptides in which it was included (see Methods for details). Figures were created using Discovery Studio Visualizer software and the crystal structure of the Cal09 HA trimeric protein PDB ID: 3LZG (10.1126/science.1186430). The HA trimeric protein is presented as a gray ribbon. Residues colored in gold comprise the given site. Residues associated with HW status are colored in blue shades according to their scores. Residues associated with obese status are colored in red shades according to their scores. Sites and scored residues are presented on a single HA subunit. Left side in each panel: Dark gold spheres represent amino acid residues belonging to each of the five regions of interest mapped onto one of the three trimeric proteins: (**A**) the HA1 subunit; (**B**) the HA2 subunit; (**C**) The fusion domain; (**D**) Receptor binding site; (**E**) The esterase domain. Right side in each Panel: Dark blue spheres represent amino acid residues within the regions of interest preferentially associated with IgG or IgA antibodies in sera from HW individuals (panels **A, C**-**D**) and dark red spheres represent amino acid residues within the regions of interest preferentially associated with IgG or IgA antibodies in sera from obese individuals (panels **A-C, E**).

Supplementary Figure 12

A)

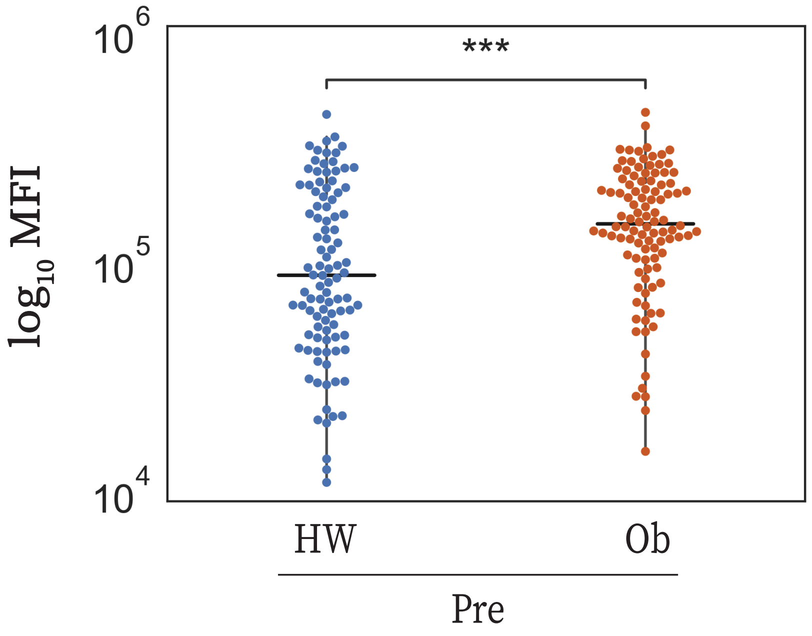

B)

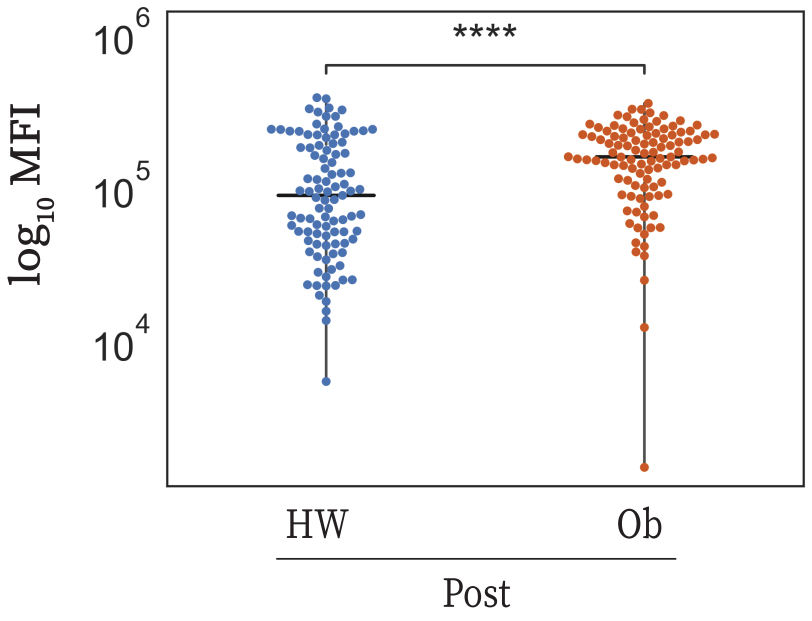

Fig S12. Binding magnitude to peptides containing the pH1N1 stalk broadly neutralizing antibodies. We used structures of 4 stalk bnAbs (C49114, CR622, F10 and FI6v3) to extract the binding epitope on the HA stalk region. We compared the binding magnitude to all peptides that contain regions of the epitope at (**A**) baseline and (**B**) post-vaccination (see methods). Statistical significance was determined using the Wilcoxon ranksum test. ** $p < 0.0001$, **** $p < 0.00001$.

Table S1. Whole inactivated viruses and recombinant HA proteins spotted on the influenza VP antigen microarrays

Subtype	Strain	Vaccine Year	Whole Virus	Recombinant Protein HA
H1N1	A/WSN/1933	-	X	X
	A/Puerto Rico/8/1934	-		X
	A/USSR/90/1977	-	X	X
	A/Brazil/11/1978	-	X	
	A/Chile/1/1983	1984-1987	X	
	A/Singapore/6/1986	1987-1997	X	
	A/Beijing/262/1995	1998-2000	X	X
	A/New Caledonia/20/1999	2000-2007	X	X
	A/Solomon Islands/3/2006	2007-2008	X	X
	A/Brisbane/59/2007	2008-2010	X	X
	A/California/7/2009 *	2010-2016	X	X
	A/Christchurch/16/2010	2010-2016	X	
H3N2	A/Bangkok/1/1979	-	X	
	A/Leningrad/360/1986	1987-1988	X	
	A/Guizhou/54/1989	-	X	X

	A/Shandong/9/1993	-	X	
	A/Sydney/5/1997	1998-2000	X	X
	A/Panama/2007/1999	2000-2004	X	
	A/New York/55/2004	2005-2006	X	X
	A/California/07/2004	-	X	
	A/Wisconsin/67/2005	2006-2008	X	X
	A/Brisbane/10/2007	2008-2010	X	X
	A/Perth/16/2009 *	2010-2012	X	X
	A/Victoria/210/2009	2011 (S)	X	X
	A/Victoria/361/2011	2012-2013	X	X
	A/Texas/50/2012	2013-2015	X	X
	A/Switzerland/9715293/2013	2015-2016	X	X
B	B/Lee/1940	-	X	
	B/Yamagata/16/1988	-	X	X
	B/Jiangsu/10/2003	2005-2006	X	
	B/Malaysia/2506/2004	2006-2008	X	X
	B/Florida/4/2006	2008-2009	X	X
	B/Brisbane/60/2008 *	2010-2017	X	X
	B/Massachusetts/2/2012	2014-2015	X	

	B/Phuket/3073/2013	2015-2016,	X	X
Number of strains:			34	23
Number of strains included in seasonal vaccines:			25	18

(S) - southern hemisphere vaccine

* The strains included in the vaccine given in this trial
